# Effectiveness and Cost Effectiveness of Expanding Harm Reduction and Antiretroviral Therapy in a Mixed HIV Epidemic: A Modeling Analysis for Ukraine

**DOI:** 10.1371/journal.pmed.1000423

**Published:** 2011-03-01

**Authors:** Sabina S. Alistar, Douglas K. Owens, Margaret L. Brandeau

**Affiliations:** 1 Department of Management Science and Engineering, Stanford University, Stanford, California, United States of America; 2 Veterans Affairs Palo Alto Health Care System, Palo Alto, California, United States of America; 3 Center for Health Policy and the Center for Primary Care and Outcomes Research, Stanford University, Stanford, California, United States of America; Johns Hopkins University, United States of America

## Abstract

**Background:**

Injection drug use (IDU) and heterosexual virus transmission both contribute to the growing mixed HIV epidemics in Eastern Europe and Central Asia. In Ukraine—chosen in this study as a representative country—IDU-related risk behaviors cause half of new infections, but few injection drug users (IDUs) receive methadone substitution therapy. Only 10% of eligible individuals receive antiretroviral therapy (ART). The appropriate resource allocation between these programs has not been studied. We estimated the effectiveness and cost-effectiveness of strategies for expanding methadone substitution therapy programs and ART in mixed HIV epidemics, using Ukraine as a case study.

**Methods and Findings:**

We developed a dynamic compartmental model of the HIV epidemic in a population of non-IDUs, IDUs using opiates, and IDUs on methadone substitution therapy, stratified by HIV status, and populated it with data from the Ukraine. We considered interventions expanding methadone substitution therapy, increasing access to ART, or both. We measured health care costs, quality-adjusted life years (QALYs), HIV prevalence, infections averted, and incremental cost-effectiveness. Without incremental interventions, HIV prevalence reached 67.2% (IDUs) and 0.88% (non-IDUs) after 20 years. Offering methadone substitution therapy to 25% of IDUs reduced prevalence most effectively (to 53.1% IDUs, 0.80% non-IDUs), and was most cost-effective, averting 4,700 infections and adding 76,000 QALYs compared with no intervention at US$530/QALY gained. Expanding both ART (80% coverage of those eligible for ART according to WHO criteria) and methadone substitution therapy (25% coverage) was the next most cost-effective strategy, adding 105,000 QALYs at US$1,120/QALY gained versus the methadone substitution therapy-only strategy and averting 8,300 infections versus no intervention. Expanding only ART (80% coverage) added 38,000 QALYs at US$2,240/QALY gained versus the methadone substitution therapy-only strategy, and averted 4,080 infections versus no intervention. Offering ART to 80% of non-IDUs eligible for treatment by WHO criteria, but only 10% of IDUs, averted only 1,800 infections versus no intervention and was not cost effective.

**Conclusions:**

Methadone substitution therapy is a highly cost-effective option for the growing mixed HIV epidemic in Ukraine. A strategy that expands both methadone substitution therapy and ART to high levels is the most effective intervention, and is very cost effective by WHO criteria. When expanding ART, access to methadone substitution therapy provides additional benefit in infections averted. Our findings are potentially relevant to other settings with mixed HIV epidemics.

*Please see later in the article for the Editors' Summary*

## Abstract 2

A cost-effectiveness study by Sabina Alistar and colleagues evaluates the effectiveness and cost effectiveness of different levels of investment in methadone, ART, or both, in the mixed HIV epidemic in Ukraine.

## Editors' Summary

**Background:**

HIV epidemics in Eastern Europe and Central Asia are mainly driven by increasing use of injection drugs combined with heterosexual transmission. In the Ukraine, in 2007, there were 82,000 officially registered people living with HIV—three times the number registered in 1999—and an estimated 395,000 HIV infected adults. The epidemic in Ukraine, like other countries in the region, is concentrated in at-risk populations, particularly people who inject drugs: in 2007, an estimated 390,000 Ukrainians were injecting drugs, an increase in drug use over the previous decade, not only in Ukraine, but in other former USSR states, owing to the easy availability of precursors for injection drugs in a climate of economic collapse.

The common practices of people who inject drugs in Ukraine and in other countries in the region, such as social injecting, syringe sharing, and using common containers, increase the risk of transmitting HIV. Public health interventions such as needle exchange can limit these risk factors and have been gradually implemented in these countries. In 2007, Ukraine approved the use of methadone substitution therapy and the current target is for 11,000 people who inject drugs to be enrolled in substitution therapy by 2011. Furthermore, since treatment for HIV-infected individuals is also necessary, national HIV control plans included a target of 90% antiretroviral therapy (ART) coverage by 2010 but in 2007 less than 10% of the 91,000 eligible people received treatment. Although the number of people who inject drugs and who receive ART is unknown, physicians are often reluctant to treat people who inject drugs using ART owing to alleged poor compliance.

**Why Was This Study Done?:**

As resources for HIV interventions in the region are limited, it is important to investigate the appropriate balance between investments in methadone substitution therapy and ART in order to maximize benefits to public health. Several studies have analyzed the cost effectiveness of methadone substitution therapy in similar settings but have not considered tradeoffs between ART and methadone substitution therapy. Therefore, to provide insights into the appropriate public health investment in methadone substitution therapy and ART in Ukraine, the researchers evaluated the public health effectiveness and cost effectiveness of different strategies for scaling up methadone substitution therapy and/or expanding ART.

**What Did the Researchers Do and Find?:**

The researchers developed a model to accommodate different population groups: people who inject drugs on substitution therapy with methadone; people who inject opiates and do not take any substitution therapy; and people who do not inject any drugs, hence do not need substitution therapy. The researchers inputted Ukraine country-level data into this model and used current HIV trends in Ukraine to make rational assumptions on possible future trends and scenarios. They considered scenarios expanding methadone substitution therapy availability, increasing acces to ART, or both. Then, the researchers measured health care costs, quality-adjusted life years (QALYs), HIV prevalence, infections averted, and incremental cost effectiveness for the different scenarios. They found that after 20 years, HIV prevalence reached 67.2% in people who inject drugs and 0.88% in people who do not inject drugs without further interventions. Offering methadone substitution therapy to 25% of people who inject drugs was the most effective strategy in reducing prevalence of HIV and was also the most cost effective, averting 4,700 infections and adding 75,700 QALYs versus the status quo at $530/QALY gained. Expanding both methadone substitution therapy and ART was also a highly cost effective option, adding 105,000 QALYs at US$1,120/QALY gained versus the methadone substitution therapy-only strategy. Offering ART to 80% of eligible people who did not inject drugs, and 10% of people who injected drugs averted only 1,800 infections, and added 76,400 QALYs at $1,330/QALY gained.

**What Do These Findings Mean?:**

The results show that methadone substitution-focused therapeutic scenarios are the most cost effective, and that benefits increase with the scale of the project, even among people who do not inject drugs. This makes a methadone substitution strategy a highly cost-effective option for addressing the growing HIV epidemic in Ukraine. Therefore, if it is not feasible to invest in large-scale methadone substitution programs for any reason, political circumstances for example, providing as much methadone substitution as is acceptable is still desirable. While substitution therapy appears to avert the most HIV infections, expanded ART provides the largest total increase in QALYs. Thus, methadone substitution therapy and ART offer complementary benefits. Because the HIV epidemic in Ukraine is representative of the HIV epidemic in Eastern Europe and Central Asia, the cost-effective strategies that the researchers have identified may help inform all decision makers faced with a mixed HIV epidemic.

**Additional Information:**

Please access these Web sites via the online version of this summary at http://dx.doi.org/10.1371/journal.pmed.1000423.

- Alliance provides information on its work supporting community action on AIDS in Ukraine
- USAID provides an HIV/AIDS Health Profile for Ukraine
- UNICEF provides information about its activities to help Ukraine fight rising HIV/AIDS infection rates
- International Harm Reduction Association provides information about the status of harm reduction interventions such as methadone substitution therapy around the world

## Introduction

The HIV epidemics in Eastern Europe and Central Asia have been rapidly growing over the past decade, mainly due to increasing injection drug use combined with heterosexual HIV transmission [1],[2]. Among the countries in the region, Ukraine's situation has raised serious concerns, since it has one of the fastest growing HIV epidemics in the world, with newly registered cases tripling between 1999 and 2007 [3]. With 82,000 officially registered cases in 2007 and an estimated 395,000 (range: 230,000–573,000) total infected adults, Ukraine's HIV prevalence is the highest in Europe [3],[4]. The HIV epidemic in Ukraine is similar to that of other countries in Eastern Europe and Central Asia [5],[6], which makes it an important case study for HIV interventions.

The epidemic in Ukraine, like other countries in the region, is still concentrated in at-risk populations, particularly injection drug users (IDUs), who represented more than 40% of newly registered infections in 2007 [3]. However, the epidemic seems to now be transitioning into the general population, raising concerns about the possibility of a generalized epidemic [7],[8]. In 1997, IDUs accounted for 83.6% of new infections; by 2007, heterosexual transmission was responsible for 38.4% of registered cases [3].

In 2007, an estimated 390,000 Ukrainians were IDUs; a large increase in drug use from the previous decade [4] has occurred not only in Ukraine but in most former USSR states, and was driven by the economic collapse in the early 1990s and the easy availability of precursors for injection drugs [7],[9]. The most common substances injected are “shirka” (liquid poppy straw) and “vint” (an ephedrine-based amphetamine) [10],[11].

Practices common among IDUs in Ukraine and other countries in the region (social injecting, syringe sharing, common containers) increase the risk of transmitting HIV [2],[10],[12]. Thus, interventions (e.g., needle exchange, bleaching, education) that can limit these risk factors have been gradually implemented [3]. In December 2007 Ukraine approved the use of methadone for substitution therapy, expanding the range of harm reduction interventions available to those treating IDUs [3],[13]. Current plans aim to enroll 11,000 IDUs in substitution therapy by 2011 [14].

Attempts have been made to increase access to antiretroviral therapy (ART) among HIV-infected individuals eligible for treatment, but progress has been limited [1],[3]. National Ukrainian HIV control plans required 90% ART coverage of those eligible for treatment by WHO criteria (CD4 cell count <350 cells/µl) by 2010 [15]. However, in 2007 only 7,700 people—less than 10% of the 91,000 eligible patients—received ART [3],[16]. The level of access to ART for HIV-infected IDUs is unknown, but reports indicate that physicians are reluctant to treat IDUs, owing to alleged poor compliance with treatment, and that police confiscate the antiretroviral medicines when arresting IDUs [11],[17]–[19].

Resources for HIV interventions in the Ukraine are limited. Hence, balancing investment between methadone substitution therapy and ART in the most effective way is important. Several studies have investigated the cost-effectiveness of methadone substitution therapy in North American and Western European settings [20]–[23], but have not considered trade-offs between ART and methadone substitution therapy. One study evaluated the cost-effectiveness of harm reduction programs in Odessa, Ukraine but did not include methadone substitution therapy as an intervention [24]. The goal of our analysis was to evaluate the effectiveness and cost-effectiveness of varying levels of investment in methadone substitution therapy, ART, or both, in Ukraine. The aim of this study is to help policymakers assess whether investments in such programs are synergistic, or whether allocating resources either to methadone substitution therapy or ART is more effective.

## Methods

### Overview

To project the evolution of a mixed HIV epidemic under different intervention strategies, we developed a dynamic compartmental model of a population of IDUs on methadone substitution therapy, IDUs injecting opiates, and non-IDUs (details in Figure S1; Table S1; Text S1). The model was parameterized using Ukraine country-level data, and calibrated against current HIV trends in Ukraine [3],[4]. Table 1 summarizes key model parameters and their sources. Individuals transition between compartments at rates determined by behavioral and epidemic characteristics in Ukraine and the natural history of HIV. We evaluated the costs and quality-adjusted life years (QALYs) associated with each scale-up strategy and performed cost-effectiveness analysis. We performed extensive sensitivity analyses on all parameters.

**Table 1:** Key parameter values, ranges, and sources.

Parameter	Value	Range	Source
**Prevalence**			
HIV prevalence IDUs	41.2%	17.3%–70.0%	[3],[4]
HIV prevalence non-IDUs	0.99%	0.73%–1.16%	Calculated
**ART**			
Access to ART – eligible non-IDUs	10%	7.0%–11.0%	[13],[16]
Access to ART – eligible IDUs	2%	0.0%–5.0%	Estimated [10],[12]
Access to ART – eligible IDUs on methadone substitution therapy	25%	0.0%–30.0%	Estimated [11],[46]
**Methadone substitution therapy program**			
Methadone substitution therapy retention, 6 mo	75%	50.0%–90.0%	[11],[46]
Percentage methadone substitution therapy “graduation”	5%	1.0%–7.0%	[11],[46]
**Injection behavior**			
Number of injections per year	250	200–300	[10],[12],[24],[33],[39]
Percent of shared injections	25%	10.0%–40.0%	[10],[12],[24],[33],[39]
Percent decrease in needle sharing due to methadone substitution therapy	85%	60.0%–99.0%	[11],[20],[38],[46]
Transmission reduction needle sharing due to ART	50%	10.0%–90.0%	[33]
**Sexual behavior**			
Number of sexual partners per year – IDUs	4.3	1.5–4.5	[24],[33]
Number of sexual partners per year – non IDUs	1.3	1–1.8	[24],[33]
Percentage sexual contacts shared by IDUs with IDUs	45%	20.0%–70.0%	[10],[12],[24],[33],[40]
Condom usage rate – IDUs not on methadone substitution therapy	40%	20.0%–60.0%	[10],[12],[24],[33],[40]
Condom usage rate – IDUs on methadone substitution therapy	45%	25.0%–65.0%	[10],[12],[24],[33],[40]
Condom usage rate – non-IDUs	45%	30.0%–70.0%	[10],[12],[24],[33],[40]
Condom effectiveness	90%	85.0%–95.0%	[23],[33],[38],[56]
Sexual transmission reduction if on ART	90%	50.0%–99.0%	[27],[33],[34]
**Annual costs (US$)**			
Non-HIV medical costs	311	200–450	[16]
HIV costs	1,200	800–1,600	Estimated [3]
ART cost - IDUs not on methadone substitution therapy + IDU services	950	750–2,500	Unpublished data and [33],[47],[48]
ART cost - IDUs on methadone substitution therapy + IDU services	750	550–2,300	Unpublished data and [33],[47],[48]
ART cost - non-IDUs	450	250–2,000	Unpublished data and [33],[47],[48]
Methadone substitution therapy regimen cost + counseling services	368	200–500	[25],[39]

### Strategies Analyzed

In 2008, only 500 of Ukraine's 400,000 IDUs received substitution therapy of any kind [3]. Hence, we assumed that no methadone substitution therapy slots were available in the comparison case, herein denoted as the status quo; this also serves as a comparison if methadone substitution therapy is unacceptable for other reasons (political, social). Reflecting Ukraine data, we assumed that baseline ART access is 10% for non-IDUs, 2% for IDUs not receiving methadone substitution therapy, and 25% for IDUs receiving methadone substitution therapy [3],[15],[16].

We analyzed incremental strategies that focused on increasing the number of methadone substitution therapy slots, expanding access to ART, or both, as summarized in Table 2.

**Table 2:** Key parameters (methadone substitution therapy slots and ART access by population) for strategies considered.

Parameter	Status Quo	Low Methadone Substitution	Moderate Methadone Substitution	High Methadone Substitution	Low Mixed	Moderate Mixed	Low ART	Moderate ART	High ART	Limited ART	High Methadone Substitution High ART
Methadone substitution therapy slots	0	500	2,000	4,000	500	500	0	0	0	0	4,000
ART access											
IDUs not on methadone substitution therapy	2%	2%	2%	2%	20%	50%	20%	50%	80%	10%	80%
IDUs on methadone substitution therapy	0%	25%	25%	25%	25%	50%	0%	0%	0%	0%	80%
Non-IDUs	10%	10%	10%	10%	20%	50%	20%	50%	80%	80%	80%

Description of strategies: Status quo, no methadone substitution therapy slots, 10% of eligible non-IDUs on ART, 2% of eligible IDUs on ART; low methadone substitution therapy, 3.1% of IDUs on methadone substitution therapy, ART according to status quo except 25% of eligible IDUs on methadone substitution therapy on ART; moderate methadone substitution therapy, 12.5% of IDUs on methadone substitution therapy, ART according to status quo except 25% of eligible IDUs on methadone substitution therapy on ART; high methadone substitution therapy, 25% of IDUs on methadone substitution therapy, ART according to status quo except 25% of eligible IDUs on methadone substitution therapy on ART; low mixed, 3.1% of IDUs on methadone substitution therapy, 20% of eligible non-IDUs and IDUs not on methadone substitution therapy on ART, 25% of IDUs on methadone substitution therapy on ART; moderate mixed, 3.1% of IDUs on methadone substitution therapy, 50% of all eligible patients on ART; low ART, no methadone substitution therapy slots, 20% of all eligible patients on ART; moderate ART, no methadone substitution therapy slots, 50% of all eligible patients on ART; high ART, no methadone substitution therapy slots, 80% of all eligible patients on ART; limited ART, no methadone substitution therapy slots, 80% of eligible non-IDUs on ART, 10% of eligible IDUs on ART; high methadone substitution therapy, high ART, 25% of IDUs on methadone substitution therapy, 80% of all eligible patients on ART.

We considered a “low methadone substitution therapy” scenario, the equivalent of Ukraine's current plans [14] scaled for our modeled population of 1,000,000 individuals (500 methadone substitution therapy slots, covering 3.1% of IDUs), a “moderate methadone substitution therapy” scenario that assumes a program that is four times larger (2,000 slots, covering 12.5% of IDUs), and a “high methadone substitution therapy” scenario (4,000 slots, reaching 25% of IDUs). We only scaled methadone substitution therapy up to 25% of IDUs because there is limited information about the practical aspects of a larger scale methadone substitution therapy program in Ukraine. Realistically it is often impractical to assume that such a program can reach a large number of IDUs effectively, since they are a marginalized population that avoids contact with authorities and the national health-care system.

For the ART strategies, we considered a “low treatment” scenario with 20% of eligible patients receiving ART [3], a “moderate treatment” scenario with 50% ART access, and a “high treatment” scenario with 80% ART access. We assumed that maximum ART access is 80% because some individuals have poor tolerance for the drugs, display poor adherence, or are diagnosed too late. For comparison, we considered a “limited treatment” scenario that provided maximum ART access (80%) to everyone except IDUs (10% access).

We considered two strategies that improved both methadone substitution therapy and ART availability: a “low mixed” scenario (500 methadone substitution therapy slots, 20% ART access), and a “moderate mixed” scenario (500 methadone substitution therapy slots, 50% ART access). Finally, we considered a “high methadone substitution therapy, high ART” strategy in which both programs would be scaled up to the highest levels we have considered.

### Antiretroviral Therapy

Following international guidelines, we assumed that patients become eligible for ART when they develop symptomatic HIV (CD4 between 200–350 cells/µl) or AIDS (CD4 cell count <200 cells/µl) [25],[26]. ART reduces viral load, which leads to lower infectivity and extended life (slower disease progression and decreased mortality in the AIDS stage) [27]–[31]. We assumed that ART reduces the probability of transmission via risky sexual contact by 90% [27]–[31]. The extent to which ART affects infectivity for equipment sharing is unknown. Similar to previous work [32],[33], we assumed a 50% reduction in the base case analysis. On the basis of published models of the natural history of HIV, we assumed that ART reduces by 6-fold the progression rate from symptomatic HIV to AIDS, and AIDS mortality by 20% [27]–[31],[34]. Evidence from Ukraine suggests that ART has had a dramatic effect on AIDS mortality [35].

### Methadone Substitution Therapy

No data are available on the effectiveness of methadone substitution therapy in Ukraine. Pilot programs using buprenorphine showed high 6-mo retention rates (75%), and decreases in IDU risky behaviors to a minimal level [11]. The doses needed for therapeutic success were lower than in other settings, possibly reflecting the decreased potency of poppy straw versus heroin [11]. Because methadone substitution therapy is at least as effective as buprenorphine [20], we assumed that results obtained using methadone substitution therapy would be similar. We only considered methadone substitution therapy as an opiate substitute, since it is more affordable for the same effectiveness and is the preferred option in countries in the region who decide to implement substitution therapy [2],[36].

We assumed that IDUs receiving methadone substitution therapy reduced equipment sharing by 85%, and had a higher likelihood of receiving ART (25% access, versus 2% access for IDUs not receiving methadone substitution therapy) [11],[20]. Since complete rehabilitation after stopping methadone substitution therapy is difficult to achieve and many individuals return to injection drug use [11], we assumed that only 5% of individuals ceasing methadone substitution therapy stopped injecting drugs.

### Model Structure

We considered a population of 1,000,000 individuals aged 15–49; most new HIV infections, as well as injection drug use, occur in this age group [3],[4]. We segmented the population into 18 compartments distinguished by drug usage status (IDUs receiving methadone substitution therapy, IDUs injecting opiates, and non-IDUs), HIV disease stage (uninfected, nonsymptomatic HIV, symptomatic HIV, or AIDS), and ART access (on treatment, not on treatment) (Table S2). Reflecting data from Ukraine, in the base case 1.6% of the adult population were IDU, with 41.2% initial HIV prevalence [3],[4]. Initial HIV prevalence in the non-IDU population was 0.99% (consistent with the overall population prevalence of 1.63%). The initial population distribution (Table 3) was computed starting from the 1,000,000 population and applying the percentages in Table 1 to obtain the fraction of individuals who are IDUs and the fraction in each disease stage.

**Table 3:** Initial population distribution for the model.

Population Group	Uninfected	Early Infection	Late Infection, Untreated	AIDS, Untreated	Late Infection, Treated	AIDS, Treated
IDUs	9,408 (0.941%)	4,944 (0.494%)	969 (0.097%)	646 (0.065%)	20 (0.0020%)	13 (0.0013%)
General population	974,292 (97.43%)	7,281 (0.728%)	1,311 (0.131%)	874 (0.087%)	146 (0.015%)	97 (0.010%)

Distribution of a population of 1,000,000 individuals: 1.6% of the total population is IDU; 75% of infected individuals are in the early HIV infection stage; 15% in the late stage; and 10% have AIDS; 10% of eligible non-IDUs and 2% of eligible IDUs are in treatment.

### Rates of Drug Use and Mortality

Individuals enter the population at age 15 uninfected with HIV in IDU compartments (2% of new entries, consistent with the proportion of the population in IDU [33]) and non-IDU compartments. We assumed that IDUs have a 1% yearly chance of spontaneously quitting drugs [37],[38]. There is no official information about the yearly incidence of drug usage in Ukraine. To model the rate of drug usage uptake, we used an annual rate of 0.0003, consistent with values used in previous analyses [23],[38] and consistent with measured HIV prevalence trends and the proportion of IDUs in the population. Individuals can die of non-AIDS–related causes (all compartments) or AIDS (AIDS compartments). Death rates from non-AIDS causes were higher for IDUs than non-IDUs, since IDUs have more risk factors (overdose, other health consequences of injection drug use). At age 49, individuals mature out of the population. The total population size decreased over time, reflecting the negative growth trend in Ukraine.

### HIV Transmission and Progression

We considered HIV transmission via sexual contacts (all individuals) and equipment sharing (IDUs). We estimated the risk of acquiring HIV from risky injections for an uninfected IDU on the basis of annual number of injections, percentage of injections involving shared equipment, the likelihood of sharing with an HIV-infected individual, and the probability of HIV transmission per risky contact. Contact infectivity depended on the disease and ART status of the infected individual; both affect viral load [32],[34]. Our parameters accounted for practices such as frontloading and backloading of syringes, needle-sharing, and shared container use, which are reported commonly among Ukrainian IDUs [10],[11],[12],[39].

For sexual transmission, we considered the average number of annual new partnerships, which is typically higher for IDUs than non-IDUs [24],[39]. We assumed random mixing in sexual partnerships, but IDUs preferentially paired with other IDUs: we assumed that 45% of the time the chosen sexual partner of an IDU is another IDU, accounting for behavior observed in Ukraine [12],[24],[40]. We estimated the annual risk of transmission to an uninfected individual per partnership on the basis of disease stage of partner, condom usage rate, and condom effectiveness.

Disease progression for infected individuals occurred at rates established by HIV natural history models [34]. Transitions between disease stages occurred at the same rate for IDUs (including those on methadone substitution therapy) and non-IDUs, but we accounted for changes caused by ART (extended life, lower mortality, decreased infectivity) [29],[31],[34].

### Model Calibration

**Figure 1:**
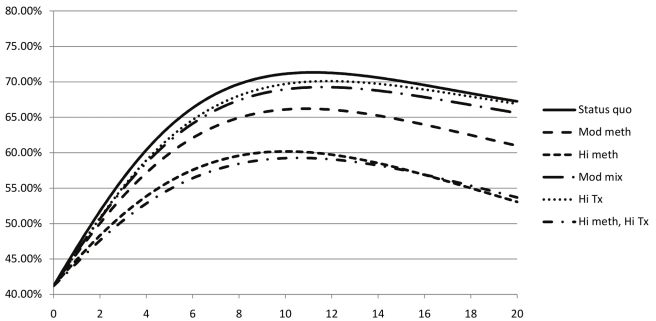
Evolution of estimated HIV prevalence among IDUs in Ukraine for various strategies to expand methadone substitution therapy and ART access. Status quo, no methadone substitution therapy slots, 10% of eligible non-IDUs on ART, 2% of eligible IDUs on ART; Mod meth, 12.5% of IDUs on methadone substitution therapy, ART according to status quo except 25% of eligible IDUs on methadone substitution therapy on ART; Hi meth, 25% of IDUs on methadone substitution therapy, ART according to status quo except 25% of eligible IDUs on methadone substitution therapy on ART; Mod mix, 3.1% of IDUs on methadone substitution therapy, 50% of all eligible patients on ART; Hi tx, no methadone substitution therapy slots, 80% of all eligible patients on ART; Hi meth, Hi tx, 25% of IDUs on methadone substitution therapy, 80% of all eligible patients on ART.

**Figure 2:**
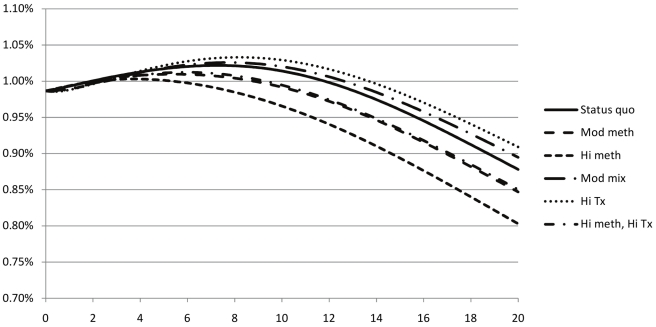
Evolution of estimated HIV prevalence among non-IDUs in Ukraine for various strategies to expand methadone substitution therapy substitution therapy and ART access. Status quo, no methadone substitution therapy slots, 10% of eligible non-IDUs on ART, 2% of eligible IDUs on ART; Mod meth, 12.5% of IDUs on methadone substitution therapy, ART according to status quo except 25% of eligible IDUs on methadone substitution therapy on ART; Hi meth, 25% of IDUs on methadone substitution therapy, ART according to status quo except 25% of eligible IDUs on methadone substitution therapy on ART; Mod mix, 3.1% of IDUs on methadone substitution therapy, 50% of all eligible patients on ART; Hi tx, no methadone substitution therapy slots, 80% of all eligible patients on ART; Hi meth, Hi tx, 25% of IDUs on methadone substitution therapy, 80% of all eligible patients on ART.

We performed several analyses to verify the outputs of our model and ensure that the population dynamics accurately reflect the trends observed in data collected from Ukraine. Without any interventions, our model predicted a slow decline in both the total population (matching the recorded demographic trends in Ukraine) and the prevalence of injection drug use. Our model predicted a sharp increase in HIV prevalence in IDUs (Figure 1), and a slower progression of HIV in the general population (Figure 2), followed by a long-term decline, since HIV-infected individuals die quickly in the absence of adequate treatment. Estimates of HIV prevalence in Ukraine vary and there is limited information about prevalence in specific risk groups. Our model was calibrated against registered total prevalence trends [3],[11]. Recent efforts to increase outreach to most-at-risk populations, including IDUs, with several prevention measures (education, condoms, needle exchange) may curb the projected increase in HIV prevalence in IDUs [3],[35].

To further verify the predictive value of our model, we initialized it with epidemic and behavioral data from mid-2005 (IDU behavioral data are not available for prior years) [3],[4],[16],[41] and compared the predicted outputs for the end of 2007 with the reported data we used to obtain the rest of the results in this paper (Table 4). Adjusting only for the lower rates of treatment availability and condom usage in 2005 [3], our model accurately matched several epidemic measures for 2.5 y later: proportion of population that is IDU, HIV prevalence in IDUs, HIV prevalence in the general population, overall incidence of HIV, and proportion of new infections occurring because of IDU versus heterosexual transmission.

**Table 4:** Model calibration, validation parameters, and sources.

Parameter	Model Estimate	Reported Value (2007)	Source
Proportion of the population that is an IDU	1.60%	1.60%	[4]
HIV prevalence among IDUs	40.88%	41.20%	[3],[4]
Overall HIV prevalence	1.62%	1.63%	[3],[4]
Yearly HIV incidence per 100,000 persons	233	190–266 (38)	Real incidence is unknown, but the officially reported numbers (in parentheses) are multiplied by a factor of 5–6 to obtain more accurate estimates.
Proportion of new infections from IDU	51.7%	51%	[3]

### Health Outcomes and Costs

For each scenario we measured costs and QALYs, discounted at 3% annually, over a 20-y time horizon. We included all costs and QALYs accruing over the time horizon as well as future discounted lifetime health-related costs and QALYs for all individuals in the population at the end of the horizon.

We assumed that quality of life decreased with HIV disease progression. IDUs had a lower quality of life than non-IDUs by a multiplicative factor of 0.9 (Table S3) [23],[33],[34],[38]. Receiving ART increased quality of life by 10% of the difference between untreated and uninfected individuals, accounting for both benefits and side effects [34],[42]–[45]. Methadone substitution therapy increased quality of life by 50% of the difference between an IDU and a non-IDU with the same disease stage and ART treatment status [38],[46].

We based our estimates of the annual costs of methadone substitution therapy on reports from Ukraine to be US$168, with US$200 in additional counseling and program support costs [33],[36]. We estimated annual ART cost to be US$450, with another US$500 for counseling and improving adherence (unpublished data) [33],[47],[48]. For individuals receiving both methadone substitution therapy and ART, we assumed that total annual counseling and program cost was US$500. All individuals incurred an annual health care cost of US$310 [16]. HIV-infected individuals incurred an additional US$1,200 in annual HIV-related health care costs [3].

## Results

Under the status quo, 33,700 new HIV infections occurred over 20 y, with 18,000 in IDUs and 15,700 in non-IDUs.

### HIV Infections Prevented

**Figure 3:**
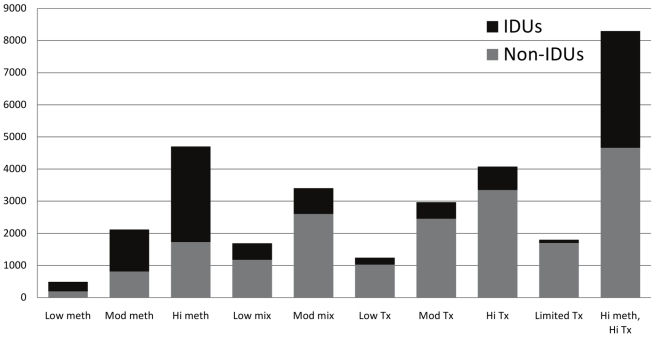
Estimated number of HIV infections averted over 20 y among IDUs and non-IDUs for strategies to expand methadone substitution therapy and ART access in Ukraine. Low meth, 3.1% of IDUs on methadone substitution therapy, ART according to status quo except 25% of eligible IDUs on methadone substitution therapy on ART; Mod meth, 12.5% of IDUs on methadone substitution therapy, ART according to status quo except 25% of eligible IDUs on methadone substitution therapy on ART; Hi meth, 25% of IDUs on methadone substitution therapy, ART according to status quo except 25% of eligible IDUs on methadone substitution therapy on ART; Low mix, 3.1% of IDUs on methadone substitution therapy, 20% of eligible non-IDUs and IDUs not on methadone substitution therapy on ART, 25% of IDUs on methadone substitution therapy on ART; Mod mix, 3.1% of IDUs on methadone substitution therapy, 50% of all eligible patients on ART; Low tx, no methadone substitution therapy slots, 20% of all eligible patients on ART; Mod tx, no methadone substitution therapy slots, 50% of all eligible patients on ART; Hi tx, no methadone substitution therapy slots, 80% of all eligible patients on ART; Limited tx, no methadone substitution therapy slots, 80% of eligible non-IDUs on ART, 10% of eligible IDUs on ART; Hi meth, hi tx, 25% of IDUs on methadone substitution therapy, 80% of all eligible patients on ART.

As expected, the “high methadone substitution therapy, high ART” scenario would avert the most infections (8,300, with 3,630 averted among IDUs and 4,670 among non-IDUs) (Figure 3; Table 5). After this, the “high methadone substitution therapy” scenario averted the most infections (4,700), with the majority (2,970) averted among IDUs. This strategy averted 1,730 infections in non-IDUs because of reductions in sexual transmission from IDUs. The “high ART” scenario averted 4,080 infections (3,350 among non-IDUs).

**Table 5:** Base case results for methadone substitution therapy and ART scale-up scenarios.

Key Parameters for Each Scenario	Status Quo	Low Methadone Substitution Therapy	Moderate Methadone Substitution Therapy	High Methadone Substitution Therapy	Low Mixed	Moderate Mixed	Low ART	Moderate ART	High ART	Limited ART	High Methadone Substitution Therapy, High ART
**HIV prevalence after 20 y**											
Overall	1.44%	1.43%	1.38%	1.29%	1.44%	1.49%	1.46%	1.50%	1.53%	1.57%	1.38%
Among IDUs	67.3%	65.8%	61.0%	53.1%	65.7%	65.6%	67.1%	67.0%	66.9%	67.1%	53.7%
Among non-IDUs	0.88%	0.87%	0.85%	0.80%	0.87%	0.89%	0.88%	0.90%	0.91%	1.00%	0.85%
**Total receiving methadone substitution therapy**	0	6,376	25,397	50,488	6,346	6,306	0	0	0	0	49,861
**Total starting ART**	6,212	6,657	7,834	9,025	16,734	31,035	16,764	31,289	39,934	22,931	36,436
**Infections averted**											
Total	0	490	2,120	4,700	1,690	3,410	1,240	2,970	4,080	1,800	8,300
Among IDUs	0	300	1,310	2,970	510	810	220	520	730	100	3,630
Among non-IDUs	0	190	810	1,730	1,180	2,600	1,020	2,450	3,350	1,700	4,670
**QALYs (1,000s)**	32,749	32,758	32,785	32,825	32,788	32,839	32,780	32,831	32,863	32,826	32,930
**Costs (US$1,000s)**	10,621,305	10,626,864	10,642,679	10,661,414	10,658,501	10,715,118	10,653,463	10,710,262	10,745,838	10,722,910	10,778,843

The effects of expansion of methadone substitution therapy scaled up with a small level of nonlinearity: the “moderate methadone substitution therapy” scenario prevented slightly more than four times as many infections as the “low methadone substitution therapy” scenario (2,120 versus 490). The “high methadone substitution therapy” scenario prevented slightly more than twice as many infections as the “moderate methadone substitution therapy” scenario (4,700 versus 2,120) owing to the increased reduction in the risk of acquiring infection from the high-prevalence IDU population. Approximately 40% of the infections averted in each scenario were among non-IDUs.

For the ART-only strategies, 80%–85% of infections averted were in non-IDUs. Since ART reduces the risk of HIV transmission but still allows a residual level of infectivity, treatment scale-up offered less than proportional benefits. The “moderate ART” scenario averted 2,970 infections, approximately 2.4 times as many as the “low ART” scenario (1,240) instead of the expected 2.5 times.

The incremental 500 methadone substitution therapy slots from “low ART” to “low mixed” (both offer ART to 20% of eligible patients, but the “mixed” scenario additionally has 500 methadone substitution therapy slots) increased the number of averted infections by 36%, from 1,240 to 1,690. More than 35% of the additional infections averted were in non-IDUs. Similarly, the “moderate mixed” scenario increased the number of averted infections versus “moderate ART” by 15%, from 2,970 to 3,410. Infections averted in the mixed scenarios were less than the sum of the individual interventions, since the same infection cannot be prevented twice for an individual who benefits (directly or indirectly) from both methadone substitution therapy and ART.

The scenario with “limited ART” access to IDUs averted only 1,800 infections (100 among IDUs), less than half the infections averted in the “high ART” scenario.

### HIV Prevalence

Under the status quo, we estimated that HIV prevalence will decrease from 1.63% to 1.44% in 20 y, because of high AIDS mortality and the natural epidemic evolution (Table 5). However, with the reported level of IDU risky behaviors and the relatively high reported condom usage [24], which limits heterosexual HIV transmission, HIV prevalence is expected to grow among IDUs from 41.2% to 67.2% (Figure 1), and decrease among non-IDUs from 0.99% to 0.88% (Figure 2).

For methadone substitution therapy-focused strategies, prevalence decreased among IDUs (Figure 1) and non-IDUs (Figure 2) in inverse proportion to the number of methadone substitution therapy slots: in the “moderate methadone substitution therapy” and “high methadone substitution therapy” scenarios, prevalence decreased, respectively, to 0.85% and 0.80% among non-IDUs, and 61.0% and 53.1% in IDUs. Because ART prolongs the lives of infected individuals, allowing them to remain longer in the population, the ART-focused strategies led to slight increases in HIV prevalence among IDUs and non-IDUs, even though HIV infections were averted.

### Cost Effectiveness

**Figure 4:**
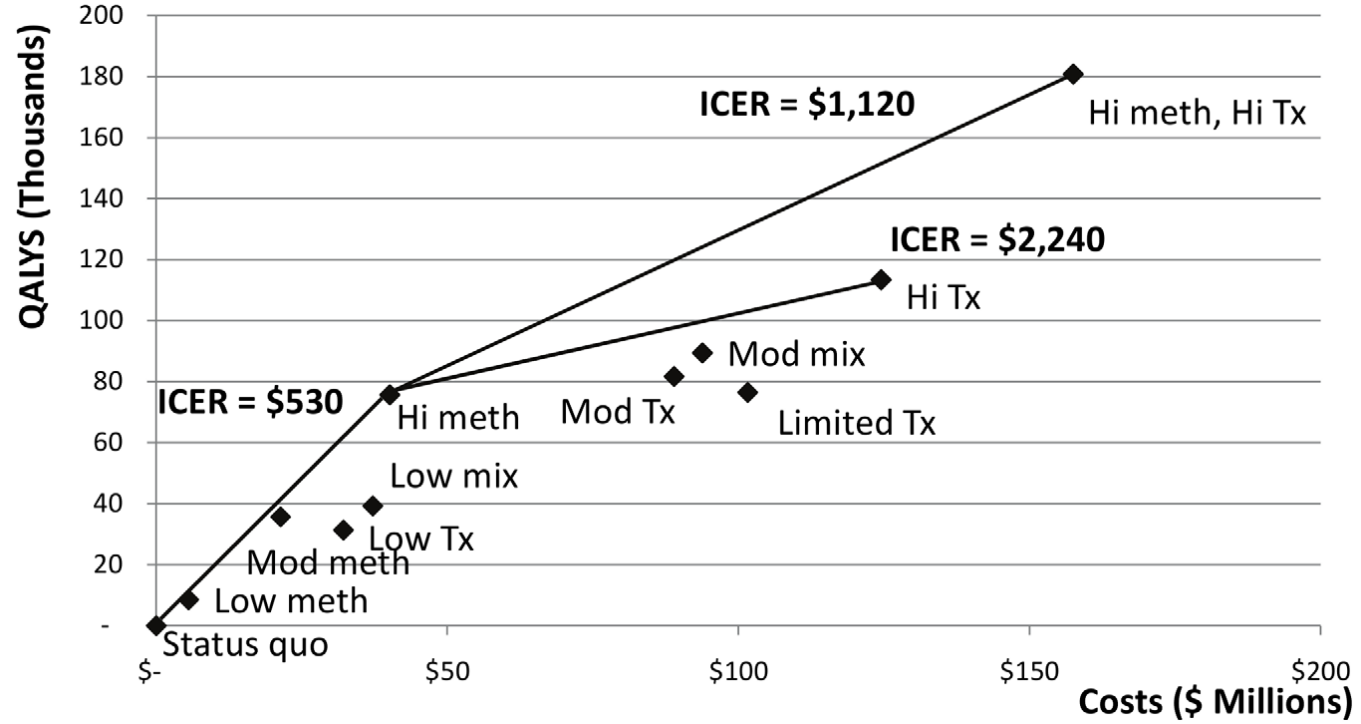
Cost effectiveness of various strategies for scaling up methadone substitution therapy and ART access in Ukraine, assuming that scaling up methadone substitution therapy is a feasible option. Status quo, no methadone substitution therapy slots, 10% of eligible non-IDUs on ART, 2% of eligible IDUs on ART; Low meth, 3.1% of IDUs on methadone substitution therapy, ART according to status quo except 25% of eligible IDUs on methadone substitution therapy on ART; Mod meth, 12.5% of IDUs on methadone substitution therapy, ART according to status quo except 25% of eligible IDUs on methadone substitution therapy on ART; Hi meth, 25% of IDUs on methadone substitution therapy, ART according to status quo except 25% of eligible IDUs on methadone substitution therapy on ART; Low mix, 3.1% of IDUs on methadone substitution therapy, 20% of eligible non-IDUs and IDUs not on methadone substitution therapy on ART, 25% of IDUs on methadone substitution therapy on ART; Mod mix, 3.1% of IDUs on methadone substitution therapy, 50% of all eligible patients on ART; Low tx, no methadone substitution therapy slots, 20% of all eligible patients on ART; Mod tx, no methadone substitution therapy slots, 50% of all eligible patients on ART; Hi tx, no methadone substitution therapy slots, 80% of all eligible patients on ART; Limited tx, no methadone substitution therapy slots, 80% of eligible non-IDUs on ART, 10% of eligible IDUs on ART; Hi meth, Hi tx, 25% of IDUs on methadone substitution therapy, 80% of all eligible patients on ART.

Due to cost differences, the methadone substitution therapy strategies were less expensive than the corresponding ART strategies, while offering significant benefits (Figure 4; Table 5). If all methadone substitution therapy scenarios are feasible in practice, the most cost-effective strategy is scaling up methadone substitution therapy as much as possible (Figure 4). The “high methadone substitution therapy” scenario costs US$530/QALY gained (confidence interval [CI] US$250–US$930/QALY gained) compared to the status quo. The most effective strategy is “high methadone substitution therapy, high ART” which costs US$1,120/QALY gained (CI US$770–US$1370/QALY gained) compared to the “high methadone substitution therapy” strategy. This strategy also has the highest total cost (Figure 4). The next most effective alternative is the “high ART” scenario, which has modestly lower total costs, but is less efficient at US$2,240/QALY gained (CI: US$940–US$4,070) compared to the “high methadone substitution therapy” strategy. According to World Health Organization (WHO) guidelines [49],[50], these strategies are all highly cost-effective because they cost less than Ukraine's per capita gross domestic product (GDP). The “mixed” scenarios and “low ART” and “moderate ART” scenarios were dominated (they cost more and generated fewer QALYs than other strategies or combinations of strategies), as was the “limited ART” scenario.

**Figure 5:**
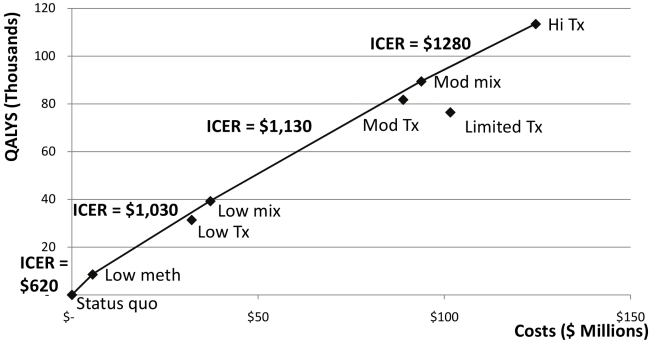
Cost effectiveness of various strategies for scaling up methadone substitution therapy and ART access in Ukraine, assuming that only a low level of methadone substitution therapy (3.1% of IDUs on methadone substitution therapy) is feasible. Status quo, no methadone substitution therapy slots, 10% of eligible non-IDUs on ART, 2% of eligible IDUs on ART; Low meth, 3.1% of IDUs on methadone substitution therapy, ART according to status quo except 25% of eligible IDUs on methadone substitution therapy on ART; Mod meth, 12.5% of IDUs on methadone substitution therapy, ART according to status quo except 25% of eligible IDUs on methadone substitution therapy on ART; Hi meth, 25% of IDUs on methadone substitution therapy, ART according to status quo except 25% of eligible IDUs on methadone substitution therapy on ART; Low mix, 3.1% of IDUs on methadone substitution therapy, 20% of eligible non-IDUs and IDUs not on methadone substitution therapy on ART, 25% of IDUs on methadone substitution therapy on ART; Mod mix, 3.1% of IDUs on methadone substitution therapy, 50% of all eligible patients on ART; Low tx, no methadone substitution therapy slots, 20% of all eligible patients on ART; Mod tx, no methadone substitution therapy slots, 50% of all eligible patients on ART; Hi tx, no methadone substitution therapy slots, 80% of all eligible patients on ART; Limited tx, no methadone substitution therapy slots, 80% of eligible non-IDUs on ART, 10% of eligible IDUs on ART; Hi meth, Hi tx, 25% of IDUs on methadone substitution therapy, 80% of all eligible patients on ART.

Given practical considerations and political reluctance to invest in programs directed to IDUs, we evaluated the cost effectiveness of the alternatives assuming that only the minimal level of methadone substitution therapy can be implemented (Figure 5). Under these constraints, the “low methadone substitution therapy” strategy is the most cost-effective alternative, with an incremental cost/QALY gained of US$650 (CI US$310–US$1,100) compared to the status quo. A more effective but more expensive option is the “low mixed” scenario (US$1,030/QALY gained, CI US$670–US$1,270), followed by the “moderate mixed” scenario (US$1,130/QALY gained, CI US$780–US$1,390), and the “high ART” scenario (US$1,280/QALY gained, CI US$810–US$1,600), all of which are considered highly cost effective by international standards [49],[50].

### Sensitivity Analyses

We performed one-way sensitivity analyses on all model parameters (Figure S2 and Table S5 summarize the key results for the “high methadone substitution therapy” scenario), and select multiway sensitivity analyses. The main parameters affecting infections averted were related to risky sexual and injecting behaviors, methadone substitution therapy effectiveness in reducing injecting-related risks, ART effectiveness in reducing infectivity, and the infectivity of unsafe sexual and equipment-sharing contacts. In all cases, the relative differences between the scenarios remained comparable to the base case, with the same alternatives being preferred. We discuss here only the most important findings from the sensitivity analyses.

We performed extensive sensitivity analysis on four key parameters (Table S4): reduction in infectivity due to ART, methadone substitution therapy effectiveness in reducing equipment sharing, IDU preference for other IDUs as sexual partners, and ART costs. We varied the reduction in sexual and injection sharing infectivity owing to ART and found that our results were generally robust. For the “high ART” scenario, if ART reduces infectivity of risky IDU contacts by 90% (base case 50%), 5,000 infections are averted (versus 4,080 in the base case). If ART only reduces sexual infectivity by 50% (base case 90%), only 1,790 infections are averted. However, these differences do not change the relative cost effectiveness of the strategies.

We varied the efficacy of methadone substitution therapy in reducing risky injection behavior. We found that “high methadone substitution therapy” prevents more infections than “high ART” if methadone substitution therapy decreases equipment sharing by at least 70%. If the decrease is 60% (versus 85% in the base case), “high methadone substitution therapy” still averts 3,230 infections, versus 4,700 in the base case. This finding is significant because information about the effectiveness of methadone substitution therapy programs in Ukraine is limited. Pilot programs with buprenorphine showed potential reductions in risky behaviors to a minimal level, and significant improvements in the quality of life of IDUs undergoing substitution therapy [11]. These data (significant reductions in risky behaviors and improvements in quality of life) are consistent with the results of other substitution therapy pilot programs in Eastern Europe (Poland, Lithuania) [46]. However, IDUs participating in the pilot programs may be the most committed to changing risky behavior, and results may change with program expansion.

Because there is uncertainty about behavioral risk parameters, we varied the percentage of sexual partners of IDUs who are also IDUs from 20% to 70% (base case 45%). We found that “high methadone substitution therapy” (which averts 4,700 infections in the base case) averts more infections than “high ART” in both cases (6,260 versus 4,960 for 20% preference and 3,380 versus 3,070 for 70% preference).

There is relatively clear information on the costs of methadone substitution therapy in Ukraine, but little information on ART costs. We varied the price of ART and related services between US$250 and US$2,000 and found that, while the total cost of ART scenarios varied, their relative rankings in terms of cost effectiveness stayed the same.

Our base case assumed constant costs per person reached by an intervention, regardless of program scale. However, as interventions scale up, the cost per person reached may increase (e.g., as programs scale up, it may become more expensive to enroll and retain individuals because of increasing outreach and adherence costs). To evaluate this possibility, in sensitivity analysis we assumed a 20% higher cost per person for scenarios involving “high methadone substitution therapy” or “high ART.” In this case, “moderate methadone substitution therapy” became slightly more cost effective (US$600/QALY gained) than “high methadone substitution therapy” (US$660/QALY gained versus “moderate methadone substitution therapy”), but both programs were highly cost effective.

To further test the robustness of our model, we performed a probabilistic sensitivity analysis (details in Text S1). We used the results from the simulation to construct 95% CIs for the number of infections averted in each strategy, and found that the mean values from the probabilistic sensitivity analysis matched the values and the trends we observed in the base case analyses (Table 6).

**Table 6:** Results of probabilistic sensitivity analysis.

Analysis	Status Quo	Low Methadone Substitution Therapy	Moderate Methadone Substitution Therapy	High Methadone Substitution Therapy	Low Mixed	Moderate Mixed	Low ART	Moderate ART	High ART	Limited ART	High Methadone Substitution Therapy, High ART
**Infections averted**											
Deterministic model	0	490	2,120	4,700	1,690	3,410	1,240	2,970	4,080	1,800	8,300
Simulation average	0	480	2,050	4,510	1,910	3,920	1,480	3,500	4,770	2,150	8,710
95% CI		280–710	1,260–3,000	2,910–6,450	1,310–2,600	2,630–5,380	950–2,130	2,290–4,940	3,120–6,680	1,290–3,140	6,240–11,480
**HIV prevalence overall**											
Deterministic model	1.44%	1.43%	1.38%	1.29%	1.44%	1.49%	1.46%	1.50%	1.53%	1.57%	1.38%
Simulation average	1.48%	1.47%	1.42%	1.33%	1.47%	1.50%	1.48%	1.51%	1.53%	1.59%	1.39%
95% CI	1.09%–1.91%	1.07%–1.89%	1.02%–1.85%	0.93%–1.76%	1.07%–1.89%	1.09%–1.92%	1.09%–1.90%	1.10%–1.93%	1.12%–1.95%	1.18%–2.04%	0.97%–1.83%
**HIV prevalence, IDUs**											
Deterministic model	67.3%	65.8%	61.0%	53.1%	65.7%	65.6%	67.1%	67.0%	66.9%	67.1%	53.7%
Simulation average	65.4%	64.0%	59.3%	51.9%	63.8%	63.5%	65.1%	64.8%	64.6%	65.2%	52.1%
95% CI	44.7%–79.4%	43.5%–78.3%	37.6%–74.7%	30.1%–68.7%	42.7%–78.1%	41.2%–78.3%	44.4%–79.4%	42.9%–79.4%	42.4%–79.6%	44.4%–79.4%	29.2%–70.1%
**QALYs (1,000s)**											
Deterministic model	32,749	32,758	32,785	32,825	32,788	32,839	32,780	32,831	32,863	32,826	32,930
Simulation average	32,849	32,857	32,884	32,923	32,891	32,945	32,883	32,937	32,971	32,930	33,036
95% CI	29,516–36,447	29,524– 36,457	29,549– 36,485	29,584– 36,524	29,543–36,500	29,578–36,568	29,535–36,492	29,572–36,560	29,600–36,602	29,566–36,550	29,663–36,664

## Discussion

Our analyses showed that the methadone substitution therapy-focused scenarios are the most cost effective, and that benefits increase with the scale of the project, even among non-IDUs. The most effective intervention is to provide high levels of methadone substitution therapy and ART, a strategy that is also economically efficient. Providing as much methadone substitution therapy as is possible is desirable, since methadone substitution therapy enhances the effects of “ART only” programs and helps prevent additional infections, even among non-IDUs. Importantly, we found that substitution therapy averted the most infections, but expanded ART along with expanded substitution therapy provided the largest total increase in QALYs. This result highlights the complementary nature of these interventions.

We analyzed the cost effectiveness of combinations of two important interventions that may potentially compete for scarce resources—harm reduction and treatment scale up. Given the importance of these interventions in controlling HIV, analyzing their use in combination and assessing the synergies between them is an important first step in making decisions about portfolios of prevention and treatment interventions in a mixed epidemic. We evaluated the cost effectiveness of these programs in Ukraine, which has one of the fastest growing HIV epidemics in the world and the highest prevalence in Europe. Its mixed epidemic is driven largely, but not exclusively, by injection drug use. The success or failure of strategies to mitigate the epidemic in Ukraine is substantial, as such strategies may be applicable to the region more broadly.

Our finding that methadone substitution therapy, even at modest levels, can substantially reduce new HIV infections and HIV prevalence is particularly significant given the importance of injection drug use in the spread of HIV in Eastern Europe, Russia, and Central Asia. Although our quantitative estimates of cost effectiveness cannot be generalized to other countries, harm reduction and substitution therapy will likely be critical to the control of HIV throughout the region.

Ukraine's decision to invest in ART and methadone substitution therapy programs could be highly economically efficient. WHO guidelines define interventions that cost less than the per capita gross domestic product (GDP) as highly cost effective [49],[50]. The per capita GDP of Ukraine was approximately US$7,000 in 2008. Hence, expansion of substitution therapy, and ART alone or in addition to methadone substitution therapy, is highly cost effective. One other study found HIV interventions targeted to IDUs in Ukraine to be cost effective [24], but their results are not comparable to ours because that study considered different interventions (provision of condoms, syringes, and information materials, peer education, but not HIV treatment), focused only in the city of Odessa, and measured the costs per infection averted from the provider's perspective.

Neglecting IDUs in national programs or providing insufficient outreach to attract and retain them in treatment programs can undermine HIV control efforts [33]. Even if 80% of eligible non-IDUs are treated, if IDUs have minimal access to ART, only half as many infections are averted among non-IDUs, because of sexual transmission from untreated IDUs. These results highlight the importance of Ukraine's commitment to address HIV among IDUs [3].

ART costs have decreased in recent years, even if the results of price negotiations have not always been fully implemented in practice (unpublished data) [47]. Further progress in lowering prices may occur if plans to involve local producers succeed. However, our analysis showed that even with an ART price of US$250, methadone substitution therapy-focused strategies have more favorable cost-effectiveness ratios than do ART-only strategies.

We estimated the benefits of methadone substitution therapy conservatively, assuming successful “graduation” was only 5%. Long-term follow-up from the buprenorphine pilot program in Ukraine is not yet available, but preliminary data suggest higher graduation rates [11], which would make methadone substitution therapy more cost effective. Additionally, we assumed that all IDUs have similar preference for IDU sexual partners, regardless of methadone substitution therapy status, which may change once individuals on methadone substitution therapy are committed to behavior changes.

While some HIV cost-effectiveness studies use disability-adjusted life years (DALYs) to measure health outcomes, we used QALYs to measure health outcomes, following recommendations for the conduct of cost-effectiveness analysis [51],[52]. QALY weights reflect quality of life in health states. DALYs were designed to evaluate the burden of disease in countries [53]–[55]. Our results are not likely to change qualitatively if DALYs are used to measure health outcomes, as both measures reflect morbidity associated with disease.

Our analysis has several limitations. We assumed homogeneous mixing between individuals in all compartments, which means that the probability of having a risky contact with an individual from a compartment depends only on the relative size of the compartment. The only exception is preferential sexual mixing by IDUs (an estimated 45% of sexual contacts are shared with other IDUs [10],[12],[24],[33],[40]). The homogeneous mixing assumption may not hold in practice, since IDUs are often involved in sexual and needle-sharing networks with other drug injectors, rather than mixing randomly. Methadone substitution therapy programs that reach IDUs central in such networks will be most effective in reducing HIV transmission. Also, we assumed that our parameters apply to the whole population, whereas in reality the epidemic characteristics vary across the country.

Data about the HIV epidemics in Ukraine, Eastern Europe, and Central Asia, in particular cost information, are limited and change rapidly. Our sensitivity analyses showed that the scenario choices are unchanged over a wide variety of parameters, but the cost-effectiveness ratios may change. More information is needed about the effectiveness of methadone substitution therapy programs in this region as well as potential economies or diseconomies of scale as methadone substitution therapy and ART programs are scaled up from their current low levels.

Our analyses evaluated the effectiveness, total expenditures, and efficiency of the strategies we considered. How policymakers choose among the alternatives we evaluated may also depend on whether there are constraints on the total budget available for such programs, which in turn depends on how policymakers prioritize interventions related to HIV relative to interventions for other health conditions, and to nonhealth spending. For example, implementation of the high levels of methadone substitution therapy and ART would require total expenditures of US$150 million over 20 y, while implementation of the high methadone substitution therapy-only strategy, although less effective, requires total expenditures of under US$50 million.

In conclusion, we have shown that methadone substitution therapy is a highly cost-effective option for addressing the growing HIV epidemic in Ukraine. A strategy that expands both methadone substitution therapy and ART to high levels is the most effective intervention, as the two interventions are complementary and synergistic. Such a strategy is very cost effective by WHO criteria. For programs that primarily expand ART, provision of minimal access to methadone substitution therapy provides additional benefit in terms of number of infections averted. Because the HIV epidemic in Ukraine is representative of the HIV epidemic in Eastern Europe and Central Asia, our analyses are relevant for decision makers faced with a mixed epidemic in this region.

## Supporting Information

Figure S1 Simplified diagram of model.
(0.77 MB DOC)

Figure S2 Changes in incremental cost-effectiveness ratio of the "high methadone substitution therapy" strategy for key parameters, with variation of parameters from low to high values.
(0.28 MB DOC)

Table S1 Summary of notation for parameters and variables.
(0.03 MB DOC)

Table S2 Characteristics of individuals in each compartment.
(0.04 MB DOC)

Table S3 Parameter values, ranges, and sources.
(0.10 MB DOC)

Table S4 Results (HIV infections averted) of select one-way sensitivity analyses on key parameters.
(0.04 MB DOC)

Table S5 Changes in incremental cost-effectiveness ratio (ICER) of the "high methadone substitution therapy" strategy compared to the status quo for the 20 parameters with the greatest influence on the ICER, ranged from low to high values.
(0.04 MB DOC)

Text S1 Appendix.
(0.21 MB DOC)

## Abbreviations

ART: antiretroviral therapy
CI: confidence interval
IDU: injection drug use
IDUs: injection drug users
QALY: quality-adjusted life year
