# Supplementary material for: Effectiveness and Cost Effectiveness of Expanding Harm Reduction and Antiretroviral Therapy in a Mixed HIV Epidemic: A Modeling Analysis for Ukraine
Source: PLoS Med. 2011 Mar 1;8(3):e1000423. doi: 10.1371/journal.pmed.1000423 (PMC3046988; doi:10.1371/journal.pmed.1000423)
Supplement: Figure S1 — Simplified diagram of model. (0.77 MB DOC) [file pmed.1000423.s001.doc]

**Figure S1**. Simplified schematic diagram of model


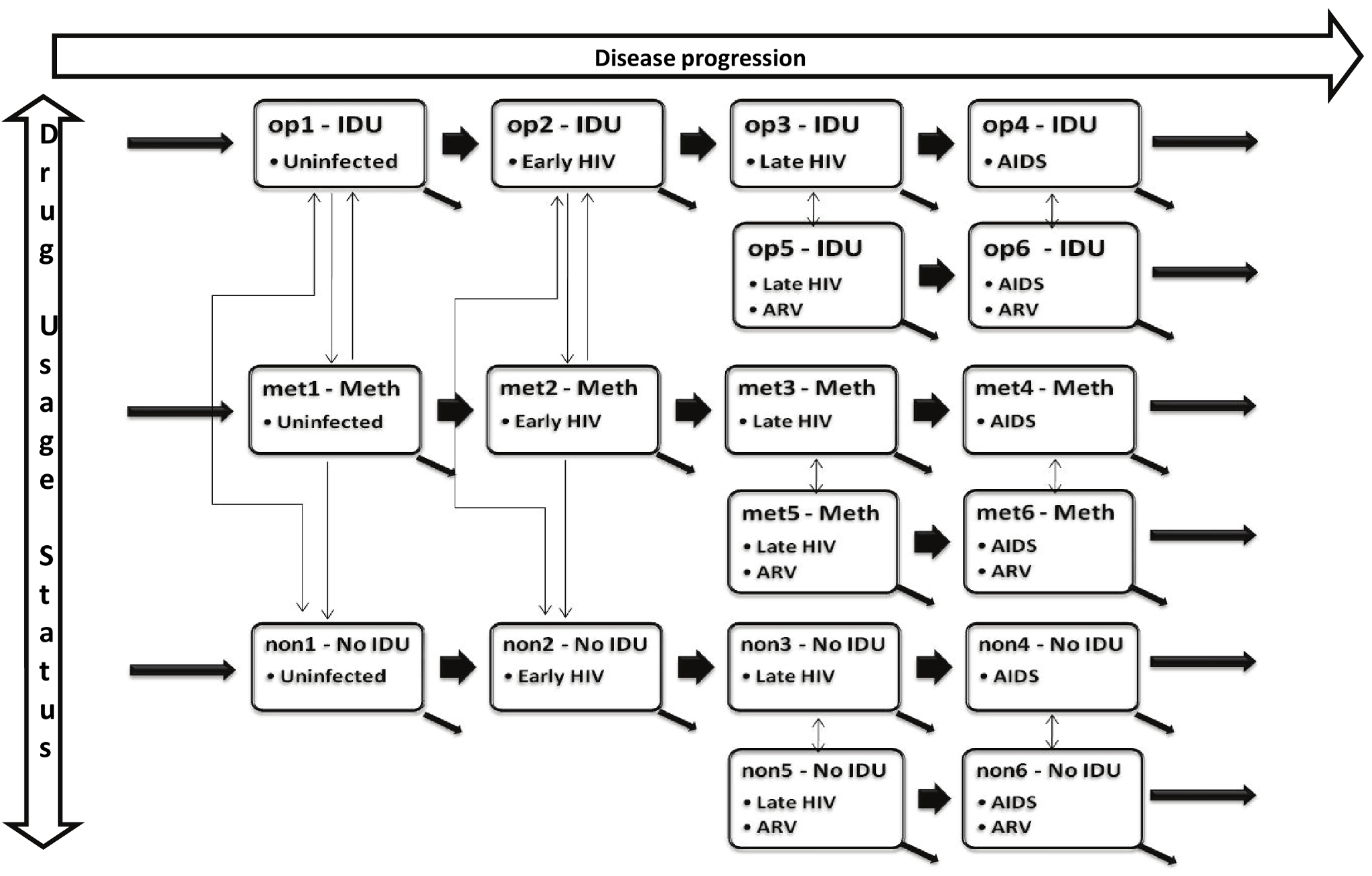


The population is divided into mutually exclusive, collectively exhaustive compartments. Each box represents a compartment. Arrows represent transitions between compartments, entry to the population through maturation, or exit from the population through death or maturation. “Early HIV” is defined as asymptomatic HIV (CD4 cell count >350 cells/µl), “Late HIV” is symptomatic HIV (CD4 cell count between 350 cells/µl and 200 cells/µl), and AIDS is defined as having a CD4 cell count< 200 cells/µl.

IDU = injection drug user, meth= methadone, ART = antiretroviral treatment
