# Supplementary material for: Effectiveness and Cost Effectiveness of Expanding Harm Reduction and Antiretroviral Therapy in a Mixed HIV Epidemic: A Modeling Analysis for Ukraine
Source: PLoS Med. 2011 Mar 1;8(3):e1000423. doi: 10.1371/journal.pmed.1000423 (PMC3046988; doi:10.1371/journal.pmed.1000423)

**Figure S2.** Changes in incremental cost-effectiveness ratio of the “high methadone” strategy for key parameters, with variation of parameters from low to high values


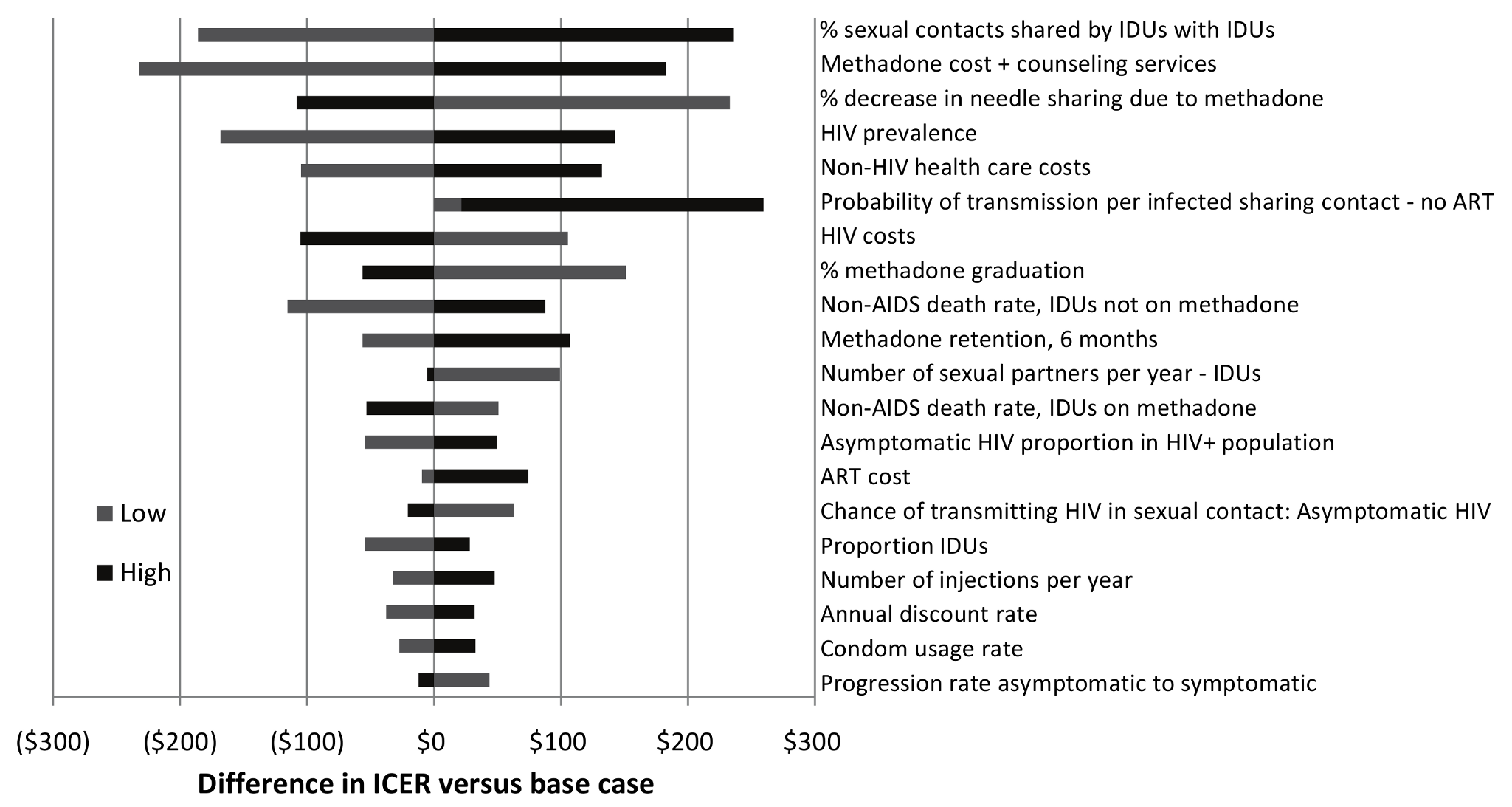

Supplement: Figure S2 — Changes in incremental cost-effectiveness ratio of the "high methadone substitution therapy" strategy for key parameters, with variation of parameters from low to high values. (0.28 MB DOC) [file pmed.1000423.s002.doc]
