# Supplementary material for: Effectiveness and Cost Effectiveness of Expanding Harm Reduction and Antiretroviral Therapy in a Mixed HIV Epidemic: A Modeling Analysis for Ukraine
Source: PLoS Med. 2011 Mar 1;8(3):e1000423. doi: 10.1371/journal.pmed.1000423 (PMC3046988; doi:10.1371/journal.pmed.1000423)
Supplement: Table S1 — Summary of notation for parameters and variables. (0.03 MB DOC) [file pmed.1000423.s003.doc]

**Table S1**. Summary of notation for parameters and variables

**Indices**

*i*, *j* Index for compartments (*i*, *j* = 1,… 6)

*t* Time index (*t*≥0)

*X*,*Y* Compartment type (*X* = “*op*” for IDUs on opiates, “*met*” for IDUs on methadone, or “*non*” for non-IDUs)

*r* Risk level of sexual contact (*r*= “L” for low, or “H” for high)

**Population parameters**

*E*(*X_i_*) Rate of entry to compartment *X_i_*

*M*(*X_i_*) Rate of maturation out of compartment *X_i_*

*D*(*X_i_*) Rate of non-AIDS death out of compartment *X_i_*

*R(op_i_)* Rate of spontaneous recovery from drug usage to return to non-IDU status out of compartment *op_i_*

*U(non_i_)* Rate of starting IDU out of compartment *non_i_*

**HIV progression parameters**

*A*(*X_i_*) AIDS death rate from compartment *X_i_* (i=4,6)

*P*(*X_i_*) Disease stage progression rate for compartment *X_i_* (*i* =2,3,5)

**ART and methadone parameters**

*H*(*X_i_*) Rate of starting (*i* =3,4) or quitting (*i* =5,6) ART

*S* Number of methadone slots available

*Q*(*met_i_*) Rate of quitting methadone (*i*=1,…6) for engaging in injection drug use

*G*(*met_i_*) Rate of graduation from methadone (*i* =1,…6) to return to non-IDU status

**Injection drug use parameters**

*I*(*X_i_*) Number of opiate injections per year for compartment *X_i_* (*X*= “*op*” or “*met*”)

*n*(*X_i_*) Percentage of shared injections for compartment X_i_ (X= “*op*” or “*met*”)

*T*(*X_i_*,*Y_j_*) Probability of transmitting HIV at risky injection contact between individual in compartment *X_i_* and individual in compartment *Y_j_*

*L*(*X_i_*,*Y_j_*) Sufficient injection-related contact rate for HIV transmission between individual in compartment *X_i_* and individual in compartment *Y_j_*

**Sexual behavior parameters**

*C*(*X_i_*,*Y_j_*) Sufficient sexual contact rate for HIV transmission between individual in compartment *X_i_* and individual in compartment *Y_j_*

*C^L^*(*X_i_*,*Y_j_*) Sufficient sexual contact rate for HIV transmission at a low risk partnership (condom was used and failed) between individual in compartment *X_i_* and individual in compartment *Y_j_*

*C^H^*(*X_i_*,*Y_j_*) Sufficient sexual contact rate for HIV transmission at a high risk partnership (condom was not used) between individual in compartment *X_i_* and individual in compartment *Y_j_*

*K*(*X_i_*) Number of sexual partners for an individual in compartment *X_i_*

*U*(*X_i_*) Condom usage rate for an individual in compartment *X_i_*

*Eff* Condom effectiveness

*ST*(*X_i_*,*Y_j_*) Probability of transmitting HIV in a risky sexual partnership between an individual in compartment *X_i_* and individual in compartment *Y_j_*

*F*(*X_i_*,*Y_j_*) Probability for an individual in compartment *X_j_* of having a sexual partnership with an individual in compartment *Y_j_*

*Aff* Percentage of sexual partners of IDUs who are also IDUs
