# Supplementary material for: Effectiveness and Cost Effectiveness of Expanding Harm Reduction and Antiretroviral Therapy in a Mixed HIV Epidemic: A Modeling Analysis for Ukraine
Source: PLoS Med. 2011 Mar 1;8(3):e1000423. doi: 10.1371/journal.pmed.1000423 (PMC3046988; doi:10.1371/journal.pmed.1000423)
Supplement: Table S2 — Characteristics of individuals in each compartment. (0.04 MB DOC) [file pmed.1000423.s004.doc]

**Table S2**. Characteristics of individuals in each compartment

| **Compartment** | **1 (***X_i_=op_1_*) | **2 (***X_i_=op_2_*) | **3 (***X_i_=op_3_*) | **4 (***X_i_=op_4_*) | **5 (***X_i_=op_5_*) | **6 (***X_i_=op_6_*) |
| --- | --- | --- | --- | --- | --- | --- |
|  | IDU, no methadone | IDU, no methadone | IDU, no methadone | IDU, no methadone | IDU, no methadone | IDU, no methadone |
| **“*op*”** | Uninfected | Asymptomatic HIV | Symptomatic HIV | AIDS | Symptomatic HIV | AIDS |
|  | No ART | No ART | No ART | No ART | ART | ART |
| **Compartment** | **1 (***X_i_=met_1_*) | **2 (***X_i_=met_2_*) | **3 (***X_i_=met_3_*) | **4 (***X_i_=met_4_*) | **5 (***X_i_=met_5_*) | **6 (***X_i_=met_6_*) |
|  | IDU on methadone | IDU on methadone | IDU on methadone | IDU on methadone | IDU on methadone | IDU on methadone |
| **“*met*”** | Uninfected | Asymptomatic HIV | Symptomatic HIV | AIDS | Symptomatic HIV | AIDS |
|  | No ART | No ART | No ART | No ART | ART | ART |
| **Compartment** | **1 (***X_i_=non_1_*) | **2 (***X_i_=non_2_*) | **3 (***X_i_=non_3_*) | **4 (***X_i_=non_4_*) | **5 (***X_i_=non_5_*) | **6 (***X_i_=non_6_*) |
|  | Non-IDU | Non-IDU | Non-IDU | Non-IDU | Non-IDU | Non-IDU |
| **“*non*”** | Uninfected | Asymptomatic HIV | Symptomatic HIV | AIDS | Symptomatic HIV | AIDS |
|  | No ART | No ART | No ART | No ART | ART | ART |
