# Supplementary material for: Effectiveness and Cost Effectiveness of Expanding Harm Reduction and Antiretroviral Therapy in a Mixed HIV Epidemic: A Modeling Analysis for Ukraine
Source: PLoS Med. 2011 Mar 1;8(3):e1000423. doi: 10.1371/journal.pmed.1000423 (PMC3046988; doi:10.1371/journal.pmed.1000423)
Supplement: Table S3 — Parameter values, ranges, and sources. (0.10 MB DOC) [file pmed.1000423.s005.doc]

**Table S3**. Parameter values, ranges and sources

| **Parameter** | **Variable Name** | **Value** | **Range** | **Source** |
| --- | --- | --- | --- | --- |
| **Population** |  |  |  |  |
| Initial population (age 15-49) |  | 1,000,000 |  |  |
| Proportion IDUs |  | 1.60% | 1.34%-1.75% | [4] |
| **Prevalence** |  |  |  |  |
| HIV prevalence IDUs |  | 41.20% | 17.3%-70.0% | [3,4] |
| HIV prevalence non-IDUs |  | 0.99% | 0.73%-1.16% | Calculated |
| **Initial disease stages** |  |  |  |  |
| Asymptomatic HIV |  | 0.75 | 0.5-1 | [33,34] |
| Symptomatic HIV |  | 0.15 | 0-0.3 | [33,34] |
| AIDS |  | 0.10 | 0-0.2 | [33,34] |
| **Entry and exit rates** |  |  |  |  |
| Entry to population | *E*(*op_1_*)+*E*(*non_1_*) | 0.030 | 0.025-0.031 | [57] |
| Percentage entry to IDU |  | 2.0% | 1.5%-2.0% | [4] |
| Maturation | *M*(*X_i_*) | 0.029 | 0.028-0.034 | [57] |
| Non-AIDS death rate, non-IDUs | *D*(*non_i_*) | 0.005 | 0.003-0.007 | [47] |
| Non-AIDS death rate, IDUs not on methadone | *D*(*op_i_*) | 0.035 | 0.02-0.05 | [23,58] |
| Non-AIDS death rate, IDUs on methadone | *D*(*met_i_*) | 0.015 | 0.009-0.021 | [23,58] |
| Rate of spontaneous IDU quitting | *R*(*op_i_*) | 0.01 | 0.005-0.015 | [38] |
| Rate of starting injection drug use | *U*(*non_i_*) | 0.0003 | 0.0002-0.0004 | Estimated [38] |
| **Annual HIV progression rates** |  |  |  |  |
| AIDS death rate, no ART | *A*(*X_4_*) | 0.517 | 0.4-0.6 | [33,59,60] |
| AIDS death rate, ART | *A*(*X_6_*) | 0.416 | 0.3-0.5 | [33,59,60] |
| Progression rate asymptomatic to symptomatic | *P*(*X_2_*) | 0.136 | 0.10-0.15 | [33] |
| Progression rate symptomatic to AIDS, no ART | *P*(*X_3_*) | 0.395 | 0.3-0.5 | [33] |
| Progression rate symptomatic to AIDS, ART | *P*(*X_5_*) | 0.062 | 0.04-0.08 | [33] |
| **ART** |  |  |  |  |
| Base case access to ART - non-IDUs | *H(non_3_*), *H(non_4_*) | 10% | 7.0%-11.0% | [13,16] |
| Base case access to ART - IDUs | *H*(*op_3_*), *H*(*op_4_*) | 2% | 0.0%-5.0% | Estimated [10,12] |
| Base case access to ART - IDUs on methadone | *H*(*met_3_*), *H*(*met_4_*) | 25% | 0.0%-30.0% | Estimated [11,46] |
| Rate of quitting ART non-IDUs | *H(non_5_*), *H(non_6_*) | 0.125 | 0.05-0.5 | Calculated [3] |
| Rate of quitting ART IDUs not on methadone | *H*(*op_5_*), *H*(*op_6_*) | 0.65 | 0.4-0.9 | Estimated [11,46] |
| Rate of quitting ART IDUs on methadone | *H*(*met_5_*), *H*(*met_6_*) | 0.40 | 0.25-0.65 | Estimated [11,46] |
| **Methadone treatment** |  |  |  |  |
| Methadone retention, 6 months | *Q*(*met_i_*) | 75% | 50.0%-90.0% | [11,46] |
| Percentage methadone “graduation” | *G*(*met_i_*) | 5% | 1.0%-7.0% | [11,46] |
| **Injection behavior** |  |  |  |  |
| Number of injections per year | *I(op_i_)* | 250 | 200-300 | [10,12,24,33,39] |
| % of shared injections | *n*(*op_i_*) | 25% | 10.0%-40.0% | [10,12,24,33,39] |
| % decrease in needle sharing due to methadone |  | 85% | 60.0%-99.0% | [11,20,38,46] |
| Transmission reduction needle sharing due to ART |  | 50% | 10.0%-90.0% | [33] |
| Probability of transmission per infected contact - no ART | *T*(*op*_1_,*Y_j_*), *T*(*met*_1_,*Y_j_*)  for *j*=2,3,4 | 0.005 | 0.0025-0.01 | [24] |
| **Sexual behavior** |  |  |  |  |
| Number of sexual partners per year - IDUs | *K*(*op_i_*), *K*(*met_i_*) | 4.3 | 1.5-4.5 | [24,33] |
| Number of sexual partners per year – non-IDUs | *K*(*non_i_*) | 1.3 | 1-1.8 | [24,33] |
| Percentage sexual contacts shared by IDUs with IDUs | *Aff* | 45% | 20.0%-70.0% | [10,12,24,33,40] |
| Condom usage rate - IDUs not on methadone | *U*(*op_i_*) | 40% | 20.0%-60.0% | [10,12,24,33,40] |
| Condom usage rate - IDUs on methadone | *U*(*met_i_*) | 45% | 25.0%-65.0% | [10,12,24,33,40] |
| Condom usage rate - non-IDUs | *U*(*non_i_*) | 45% | 30.0%-70.0% | [10,12,24,33,40] |
| Condom effectiveness | *Eff* | 90% | 85.0%-95.0% | [23,33,38,56] |
| Sexual transmission reduction if on ART |  | 90% | 50.0%-99.0% | [27,33,34] |
| **Chance of transmitting HIV per sexual partnership** |  |  |  |  |
| Asymptomatic HIV | *ST*(*X*_1_,*Y_2_*) | 0.04 | 0.01-0.05 | Estimated [24,33] |
| Symptomatic HIV, no ART | *ST*(*X*_1_,*Y_3_*) | 0.05 | 0.02-0.07 | Estimated [24,33] |
| AIDS, no ART | *ST*(*X*_1_,*Y_4_*) | 0.08 | 0.05-0.11 | Estimated [24,33] |
| Symptomatic HIV, ART | *ST*(*X*_1_,*Y_5_*) | 0.005 | 0.007-0.01 | Calculated |
| AIDS, ART | *ST*(*X*_1_,*Y_6_*) | 0.008 | 0.0011-0.025 | Calculated |
| **Quality adjustments** |  |  |  |  |
| IDU, no methadone, no HIV |  | 0.90 | 0.8-1.0 | [23,33,34,38,61] |
| IDU, no methadone, asymptomatic HIV |  | 0.85 | 0.75-1.0 | [23,33,34,38,61] |
| IDU, no methadone, symptomatic HIV |  | 0.73 | 0.65-0.77 | [23,33,34,38,61] |
| IDU, no methadone, AIDS |  | 0.63 | 0.56-0.72 | [23,33,34,38,61] |
| Methadone, no HIV |  | 0.95 | 0.84-1.00 | Calculated |
| Methadone, asymptomatic HIV |  | 0.90 | 0.75-1.00 | Calculated |
| Methadone, symptomatic HIV |  | 0.77 | 0.65-0.80 | Calculated |
| Methadone, AIDS |  | 0.67 | 0.57-0.75 | Calculated |
| Non-IDU, no HIV |  | 1 | 0.9-1.0 | [23,33,34,38,61] |
| Non-IDU, asymptomatic HIV |  | 0.94 | 0.85-1.0 | [23,33,34,38,61] |
| Non-IDU, symptomatic HIV |  | 0.81 | 0.70-0.90 | [23,33,34,38,61] |
| Non-IDU, AIDS |  | 0.70 | 0.60-0.80 | [23,33,34,38,61] |
| Percentage increase if on methadone |  | 50% | 0.3-0.7 | [20,21,22,38,62] |
| Percentage increase if on ART |  | 10% | 0.0-0.3 | [34,42,43,44,45] |
| **Annual costs** |  |  |  |  |
| Discount rate |  | 3% | 0%-5% | [49] |
| Non-HIV health care costs |  | $311 | $200-$450 | [16] |
| HIV costs |  | $1,200 | $800-$1600 | Estimated [3] |
| ART cost IDUs not on methadone + IDU services |  | $950 | $750-$2500 | Unpublished data and [33,47,48] |
| ART cost IDUs on methadone + IDU services |  | $750 | $550-$2300 | Unpublished data and [33,47,48] |
| ART cost non-IDUs |  | $450 | $250-$2000 | Unpublished data and [33,47,48] |
| Methadone cost + counseling services |  | $368 | $200-$500 | [33,36] |
