# Supplementary material for: Effectiveness and Cost Effectiveness of Expanding Harm Reduction and Antiretroviral Therapy in a Mixed HIV Epidemic: A Modeling Analysis for Ukraine
Source: PLoS Med. 2011 Mar 1;8(3):e1000423. doi: 10.1371/journal.pmed.1000423 (PMC3046988; doi:10.1371/journal.pmed.1000423)
Supplement: Table S4 — Results (HIV infections averted) of select one-way sensitivity analyses on key parameters. (0.04 MB DOC) [file pmed.1000423.s006.doc]

**Table S4. Results (HIV infections averted) of select one-way sensitivity analyses on key parameters**

| **Scenario** | **Low Meth-adone** | **Moderate Meth-adone** | **High Meth-adone** | **Low Mixed** | **Moderate Mixed** | **Low Treat-ment** | **Moderate Treat- ment** | **High Treat-ment** | **Limited Treat-ment** | **High Meth-adone, High Treat-ment** |
| --- | --- | --- | --- | --- | --- | --- | --- | --- | --- | --- |
| Base case | 490 | 2,120 | 4,700 | 1,700 | 3,410 | 1,240 | 2,970 | 4,080 | 1,800 | 8,300 |
| ART needle transmission reduction = 10% | 470 | 2,040 | 4,550 | 1,430 | 2,830 | 1,000 | 2,410 | 3,280 | 1,680 | 7,300 |
| ART needle transmission reduction = 90% | 510 | 2,190 | 4,860 | 1,960 | 4,050 | 1,500 | 3,590 | 5,000 | 1,920 | 9,430 |
| % decrease in needle sharing due to methadone = 60% | 360 | 1,510 | 3,230 | 1,560 | 3,280 | 1,240 | 2,970 | 4,080 | 1,800 | 6,910 |
| % decrease in needle sharing due to methadone = 99% | 560 | 2,470 | 5,610 | 1,760 | 3,480 | 1,240 | 2,970 | 4,080 | 1,800 | 9,150 |
| Sexual transmission reduction from ART = 50% | 460 | 2,010 | 4,490 | 1,000 | 1,734 | 564 | 1,300 | 1,790 | 5,70 | 5,930 |
| Sexual transmission reduction from ART = 99% | 500 | 2,140 | 4,750 | 1,840 | 3,770 | 1,390 | 3,330 | 4,570 | 2,060 | 8,810 |
| % sexual contacts shared by IDUs with IDUs = 20% | 660 | 2,840 | 6,260 | 2,130 | 4,200 | 1,530 | 3.620 | 4,960 | 2,080 | 10,400 |
| % sexual contacts shared by IDUs with IDUs = 70% | 350 | 1,510 | 3,380 | 1,240 | 2,550 | 920 | 2,230 | 3,070 | 1,480 | 6,230 |
