# Supplementary material for: Effectiveness and Cost Effectiveness of Expanding Harm Reduction and Antiretroviral Therapy in a Mixed HIV Epidemic: A Modeling Analysis for Ukraine
Source: PLoS Med. 2011 Mar 1;8(3):e1000423. doi: 10.1371/journal.pmed.1000423 (PMC3046988; doi:10.1371/journal.pmed.1000423)
Supplement: Table S5 — Changes in incremental cost-effectiveness ratio (ICER) of the "high methadone substitution therapy" strategy compared to the status quo for the 20 parameters with the greatest influence on the ICER, ranged from low to high values. (0.04 MB DOC) [file pmed.1000423.s007.doc]

**Table S5. Changes in incremental cost-effectiveness ratio (ICER) of the “high methadone” strategy compared to the status quo for the 20 parameters with the greatest influence on the incremental cost-effectiveness ratio (ICER), ranged from low to high values**

|  | **ICER ($/QALY gained) if parameter value is:** | |  |
| --- | --- | --- | --- |
| **Parameter** | **Low** | **High** | **Difference** |
| Percentage sexual contacts shared by IDUs with IDUs | 344 | 766 | 422 |
| Methadone cost + counseling services | 298 | 712 | 415 |
| Percentage decrease in needle sharing due to methadone | 763 | 422 | 341 |
| HIV prevalence | 362 | 673 | 311 |
| Non-HIV health care costs | 425 | 662 | 237 |
| Probability of transmission per infected sharing contact - no ART | 552 | 768 | 216 |
| HIV costs | 635 | 425 | 210 |
| Percentage methadone “graduation” | 681 | 474 | 207 |
| Non-AIDS death rate, IDUs not on methadone | 415 | 618 | 203 |
| Methadone retention, 6 months | 474 | 637 | 163 |
| Number of sexual partners per year - IDUs | 629 | 525 | 105 |
| Non-AIDS death rate, IDUs on methadone | 581 | 477 | 104 |
| Asymptomatic HIV proportion in HIV+ population | 476 | 580 | 104 |
| ART cost | 520 | 604 | 84 |
| Chance of transmitting HIV in sexual contact: Asymptomatic HIV | 593 | 509 | 84 |
| Proportion IDUs | 476 | 558 | 82 |
| Number of injections per year | 498 | 578 | 80 |
| Annual discount rate | 492 | 562 | 70 |
| Condom usage rate | 503 | 563 | 60 |
| HIV progression rate from asymptomatic to symptomatic | 574 | 518 | 56 |
