# Supplementary material for: Effectiveness and Cost Effectiveness of Expanding Harm Reduction and Antiretroviral Therapy in a Mixed HIV Epidemic: A Modeling Analysis for Ukraine
Source: PLoS Med. 2011 Mar 1;8(3):e1000423. doi: 10.1371/journal.pmed.1000423 (PMC3046988; doi:10.1371/journal.pmed.1000423)
Supplement: Text S1 — Appendix. (0.21 MB DOC) [file pmed.1000423.s008.doc]

**Effectiveness and cost effectiveness of expanding harm reduction and antiretroviral therapy in a mixed HIV epidemic:**

**An analysis for Ukraine**

**Running head: Harm reduction and ART in Ukraine**

Sabina S. Alistar, M.S.^1^

Douglas K. Owens, M.D., M.S.^2,3^

Margaret L. Brandeau, Ph.D.^1^

January 15, 2011

^1^ Department of Management Science and Engineering, Stanford University, Stanford, CA, USA

^2^ Veterans Affairs Palo Alto Health Care System, Palo Alto, CA, USA

^3^ Center for Health Policy and the Center for Primary Care and Outcomes Research, Stanford University, Stanford, CA, USA

Correspondence to Sabina S. Alistar, Stanford University, PO Box 17244, Stanford, California, 94309, USA.

E-mail: ssabina@stanford.edu

**APPENDIX**

**Overview**

Figure S1 provides a schematic representation of the model. Figure S2 summarizes sensitivity analysis results for the 20 parameters that have the greatest effect on the cost-effectiveness ratio of the “high methadone” scenario. Table S1 summarizes model notation. For each compartment, *X_i_*(*t*) represents the number of individuals in compartment *X*, with disease and treatment status *i*, at time *t*. Table S2 details model compartments. Table S3 shows values, ranges, and sources for parameters, along with parameter names. Transitions between compartments, and into or out of the population, occur at rates defined by demographic parameters, disease progression parameters, and resource availability (ART, methadone). All rates were estimated on a yearly basis. Table S4 summarizes the results of key sensitivity analyses in terms of infections averted for the most important model parameters. Table S5 presents the values of the cost-effectiveness ratio for the “high methadone” scenario for the 20 parameters that have the greatest effect on the cost-effectiveness ratio of the this scenario.

**Population dynamics**

Individuals enter the model at age 15 (14 year olds turning 15) at rate *E*(*X_i_*) (for compartments *op_1_* or *non_1_*). The total rate of maturation into the population is *E*(*op_1_*)+*E*(*non_1_*). The ratio *E*(*op_1_*)/ *E*(*op_1_*)+*E*(*non_1_*) is equal to the percentage of IDUs in the population. We assumed that all new entrants to the population are uninfected with HIV, hence entry occurs only in compartments *op_1_* and *non_1_* . Individuals may leave the system if they die of non-AIDS related causes at rates *D*(*X_i_*) from all compartments, or AIDS at rate *A*(*X_i_*) only from compartments *X_4_* and *X_6_*. AIDS deaths occur only from compartments *X_4_* and *X_6_*. At age 49, individuals mature out of the population at rate *M*(*X_i_*) from all compartments. Values for all parameters are listed in Table S3. Entry rates are as follows:

*E*(*op_1_*)+*E*(*non_1_*)=0.03

*E*(*op_1_*)=0.02*0.03=0.0006

*E*(*non_1_*)=0.98*0.03=0.0294

*E*(*X_i_*)=0 for all *X*≠ *op_1_* or *non_1_*.

**Disease progression**

Once infected, individuals progress through the disease stages according to rates *P*(*X_i_*), listed in Table S3. These rates are computed as the reciprocal of the typical time spent in each stage according to models of the natural history of HIV. ART lowers the progression rates *P*(*X_i_*) by increasing the time spent in each stage, and also lowering the AIDS death rate *A*(*X_i_*). Thus, the AIDS death rate *A*(*X_4_*) corresponding to the untreated AIDS compartments is higher than the AIDS death rate *A*(*X_6_*) in treated compartments*.* Drug usage status and methadone treatment do not affect the evolution of the disease. Individuals become more infectious as they progress in the disease.

**HIV transmission**

In computing the rates of HIV transmission we took an approach similar to Long et al. [33]. Non-IDUs can acquire HIV only through risky sexual contacts with infected individuals. Condom usage (40% for IDUs on opiates, 45% for non-IDUs and IDUs on methadone) reduces the riskiness of sexual contacts, but is only 90% effective. IDUs can get infected either through risky sexual contacts or by sharing injection equipment (25% of injections are with shared equipment) with an infected individual. The number of individuals in compartments *X_1_* who acquire HIV at any time *t* is a function of the sufficient contact rates with infected individuals. The sufficient contact rates for risky sexual or equipment sharing encounters between individuals are computed as the product between the number of risky contacts of each kind and the probability that the contact is with an individual in an infected compartment.

The sufficient needle sharing rate (i.e., the sharing rate sufficient to transmit HIV infection) for contacts between an uninfected IDU in compartment *X_i_* and infected IDU in compartment *Y_j_* is defined only for compartments *op_1_* and *met_1_* (which contain uninfected IDUs) and is computed as:

$$L(X_{i,}Y_{j})=I(X_{i})\times n(X_{i})\times T(X_{i},Y_{j})\times\left[\frac{Y_{j}(t)\times I(Y_{j})\times n(Y_{j})}{\sum_{q=1}^{6} op_{q}(t)\times I(op_{q})\times n(op_{q})+\sum_{q=1}^{6} met_{q}(t)\times I(met_{q})\times n(met_{q})}\right]$$

where

*I*(*op_i_*)**n*(*op_i_)*=250*25%=62.5;

*I(met_i_*)**n*(*met_i_*)=(100%-85%)*250*25%=9.4;

*T*(*op_1_*,*Y_2_*)= *T*(*op_1_*,*Y_3_*)= *T*(*op_1_*,*Y_4_*)= *T*(*met_1_*,*Y_2_*)= *T*(*met_1_*,*Y_3_*)= *T*(*met_1_*,*Y_4_*)=0.005;

*T*(*op_1_*,*Y_5_*)= *T*(*op_1_*,*Y_6_*)= *T*(*met_1_*,*Y_5_*)= *T*(*met_1_*,*Y_6_*)=0.005*50%=0.0025.

The sufficient needle sharing rate is obtained by multiplying the number of shared injections with the probability of sharing them with an individual from infected compartment *Y_j_*, and the probability that the infection gets transmitted for the risky contact.

A similar approach is taken for computing sufficient sexual contact rates *C*(*X_i_*,*Y_j_*): we multiply the number of risky partnerships with the chance of acquiring HIV infection per risky partnership and the probability that the sexual contact is with an infected individual. Sufficient sexual contact rates are defined for all uninfected compartments (*op_1_*, *met_1_* and *non_1_*). We considered two types of risky sexual contacts, depending on whether a condom was used and was ineffective (low-risk contact) or was not used at all (high-risk contact). We have

$$C(X_{i},Y_{j})=C^{L}(X_{i},Y_{j})+C^{H}(X_{i},Y_{j})$$

$$C^{L}(X_{i,}Y_{j})=K(X_{i})\times U(X_{i})\times(1-Eff)\times ST(X_{i},Y_{j})\times F(X_{i},Y_{j})$$

$$C^{H}(X_{i,}Y_{j})=K(X_{i})\times(1-U(X_{i}))\times ST(X_{i},Y_{j})\times F(X_{i},Y_{j})$$

The above rates are obtained by multiplying the number of yearly sexual partners with the probability of using a condom and failing (low risk) or the probability of not using a condom (high risk), the probability of acquiring HIV per partnership, and the probability *F*(*X_i_*,*Y_j_*) of encountering a sexual partner from compartment *Y_j_*.

The probability *F*(*X_i_*,*Y_j_*) is computed as described below, assuming that IDUs prefer other IDUs as sexual partners and thus 45% of their sexual contacts are with other IDUs. The remaining contacts must be with non-IDUs.

$$F(X_{i},Y_{j})=Aff\times\frac{K(Y_{j})\times Y_{j}(t)}{\sum_{q=1}^{6} op_{q}(t)\times K(op_{q})+\sum_{q=1}^{6} met_{q}(t)\times K(met_{q})}; X,Y\in\left\{op,met\right\}$$

$$F(X_{i},Y_{j})=(1-Aff)\times\frac{K(Y_{j})\times Y_{j}(t)}{\sum_{q=1}^{6} non_{q}(t)\times K(non_{q})}; X\in\left\{op,met\right\},Y=non$$

$$F(X_{i},Y_{j})=(1-Aff)\times\frac{K(Y_{j})\times Y_{j}(t)}{\sum_{q=1}^{6} non_{q}(t)\times K(non_{q})}; X=non,Y\in\left\{op,met\right\}$$

$$F(X_{i},Y_{j})=1-(1-Aff)\times\frac{K(Y_{j})\times Y_{j}(t)}{\sum_{q=1}^{6} non_{q}(t)\times K(non_{q})}; X=non,Y=non$$

For any given compartment *X_i_* the above probabilities sum up to 1 over all *Y_j_*.

**ART availability**

ART is available to individuals whose disease has advanced to symptomatic HIV and AIDS (CD4 cell count below 350 cells/µl). In our model, compartments *X_3_*, *X_4_*, *X_5_* and *X_6_* contain individuals eligible for treatment. At time *t*, a fraction *H*(*X_i_*) of individuals in compartment *X_i_*(*t*) enter treatment for i = 3,4 or exit treatment for i = 5,6. *H*(*X_i_*) varies by IDU status (higher for non-IDUs and IDUs on methadone than for IDUs not on methadone) and is also modified in scenarios involving increased access to treatment (Table 2). When treatment is started, the individual transitions to the corresponding “treatment” compartment (*X_3_* to *X_5_*, *X_4_* to *X_6_*). When exiting treatment, individuals transition to the corresponding “untreated” compartment (from *X_5_* to *X_3_*, and from *X_6_* to *X_4_)*.

In addition to affecting the disease progression rates *P*(*X_i_*) and the AIDS death rates *A*(*X_i_*), treatment reduces infectivity for individuals receiving it, both for sexual transmission (by 90%) and equipment sharing transmission (by 50%). Thus, if the chance of acquiring HIV from a sexual partnership with an untreated individual with symptomatic HIV is 0.05, treatment reduces the rate to 0.005. The rate for an individual with AIDS is reduced from 0.08 to 0.008. The rate of acquiring HIV from a risky contact involving equipment sharing is 0.005 if the contact individual is untreated, and 0.0025 if the contact is receiving ART.

**Methadone access**

Methadone substitution therapy is available for IDUs. When starting methadone therapy, individuals move from compartment *op_i_* to the corresponding compartment *met_i_*. IDUs can receive no intervention (*op_i_* for i=1,..4), only methadone (*met_i_* for i= 1,..4), only ART (*op_i_* for i=5,6) or both (*met_i_* for i=5,6). We assumed a fixed total number of methadone slots S is available and that all slots are in use at all times. Thus,

$\sum_{i=1}^{6} met_{i}(t)$= S.

Although the total number of methadone slots has the hard constraint S, at any time *t*, the slots need to be allocated between IDUs at different disease stages. We computed the necessary split of methadone slots at time *t* based on the distribution of IDUs in the different disease stages at time *t*-1, since we assumed recruitment into the methadone programs will reflect the epidemic dynamic in the general IDU population:

*met_i_*(*t*)= $S\times\left[\frac{op_{i}(t-1)}{\sum_{i=1}^{6} op_{i}(t-1)}\right]$

Starting with *met_i_*(*t*-1), we first computed all the changes occurring due to known rates of transition (successful methadone graduation, IDUs quitting methadone, deaths). We then “recruited” enough IDUs from the corresponding *op* compartments in order to fill up the available methadone slots up to the *met_i_*(*t*) determined from the equation above. Thus, the necessary number of “new recruits” for each *met* compartment at time *t* is computed as:

$\Delta met_{i}$(*t*)= *met_i_*(*t*)- *met_i_*(*t*-1)

IDUs in methadone treatment have lower non-AIDS death rates *D*(*met_i_*)=0.015 than IDUs who inject opiates *D*(*op_i_*)=0.035, slightly higher condom usage rates (45% versus 40%), a reduction in the percentage of shared injections (by 85%) and, depending on the strategy considered, sometimes higher access to ART.

**Model equations**

The model comprises a system of non-linear differential equations. The equations governing the change in number of individuals in each compartment over time are presented below.

Change in time for uninfected IDUs on methadone:

$$\frac{dmet_{1}(t)}{dt}=-met_{1}(t)\left[M(met_{1})+D(met_{1})+Q(met_{1})+G(met_{1})+\sum_{j=2}^{6} \sum_{Y\in\left\{op,met,non\right\}} (L(met_{1},Y_{j})(t)+C(met_{1},Y_{j})(t))\right]+\Delta met_{1}(t)$$

Change in time for uninfected IDUs not on methadone:

$$\begin{matrix} \frac{dop_{1}(t)}{dt}=E(op_{1})-op_{1}(t)\left[M(op_{1})+D(op_{1})+\sum_{j=2}^{6} \sum_{Y\in\left\{op,met,non\right\}} (L(op_{1},Y_{j})(t)+C(op_{1},Y_{j})(t))\right] \\ -\frac{dmet_{1}(t)}{dt}+met_{1}(t)\times Q(met_{1}) \end{matrix}$$

Change in time for uninfected non-IDUs:

$$\frac{dnon_{1}(t)}{dt}=E(non_{1})-non_{1}(t)\left[M(non_{1})+D(non_{1})+\sum_{j=2}^{6} \sum_{Y\in\left\{op,met,non\right\}} C(non_{1},Y_{j})(t)\right]+met_{1}(t)\times G(met_{1})$$

Similar equations describe the dynamics for the other 15 model compartments, with the only difference that entry occurs from other compartments instead of through aging into the population, and individuals progress in the disease instead of acquiring it. These equations are as follows.

Change in time for IDUs with asymptomatic HIV not receiving methadone:

$$\begin{matrix} \frac{dop_{2}(t)}{dt}=op_{1}(t)\left[\sum_{j=2}^{6} \sum_{Y\in\left\{op,met,non\right\}} (L(op_{1},Y_{j})(t)+C(op_{1},Y_{j})(t))\right]-op_{2}(t)\left[M(op_{2})+D(op_{2})+P(op_{2})\right] \\ -\frac{dmet_{2}(t)}{dt}+met_{2}(t)\times Q(met_{2}) \end{matrix}$$

Change in time for IDUs with symptomatic HIV not on methadone not receiving ART:

$$\begin{matrix} \frac{dop_{3}(t)}{dt}=op_{2}(t)\times P(op_{2})-op_{3}(t)\left[M(op_{3})+D(op_{3})+P(op_{3})+H(op_{3})\right]-\frac{dmet_{3}(t)}{dt} \\ +met_{3}(t)\times Q(met_{3})+op_{5}(t)\times H(op_{5}) \end{matrix}$$

Change in time for IDUs with AIDS not on methadone not receiving ART:

$$\begin{matrix} \frac{dop_{4}(t)}{dt}=op_{3}(t)\times P(op_{3})-op_{4}(t)\left[M(op_{4})+D(op_{4})+A(op_{4})+H(op_{4})\right]-\frac{dmet_{4}(t)}{dt} \\ +met_{4}(t)\times Q(met_{4})+op_{6}(t)\times H(op_{6}) \end{matrix}$$

Change in time for IDUs with symptomatic HIV, not receiving methadone but on ART:

$$\frac{dop_{5}(t)}{dt}=op_{3}(t)\times H(op_{3})-op_{5}(t)\left[M(op_{5})+D(op_{5})+P(op_{5})+H(op_{5})\right]-\frac{dmet_{5}(t)}{dt}+met_{5}(t)\times Q(met_{5})$$

Change in time for IDUs with AIDS not on methadone but receiving ART:

$$\begin{matrix} \frac{dop_{6}(t)}{dt}=op_{5}(t)\times P(op_{5})-op_{6}(t)\left[M(op_{6})+D(op_{6})+A(op_{6})+H(op_{6})\right]-\frac{dmet_{6}(t)}{dt} \\ +met_{6}(t)\times Q(met_{6})+op_{4}(t)\times H(op_{4}) \end{matrix}$$

Change in time for IDUs on methadone with asymptomatic HIV:

$$\begin{matrix} \frac{dmet_{2}(t)}{dt}=met_{1}(t)\left[\sum_{j=2}^{6} \sum_{Y\in\left\{op,met,non\right\}} (L(met_{1},Y_{j})(t)+C(met_{1},Y_{j})(t))\right] \\ -met_{2}(t)\left[M(met_{2})+D(met_{2})+Q(met_{2})+G(met_{2})+P(met_{2})\right]+\Delta met_{2}(t) \end{matrix}$$

Change in time for IDUs on methadone with symptomatic HIV but no ART:

$$\begin{matrix} \frac{dmet_{3}(t)}{dt}=met_{2}(t)\times P(met_{2})-met_{3}(t)\left[M(met_{3})+D(met_{3})+Q(met_{3})+G(met_{3})+P(met_{3})+H(met_{3})\right] \\ +\Delta met_{3}(t)+met_{5}(t)\times H(met_{5}) \end{matrix}$$

Change in time for IDUs on methadone with AIDS but no ART:

$$\begin{matrix} \frac{dmet_{4}(t)}{dt}=met_{3}(t)\times P(met_{3})-met_{4}(t)\left[M(met_{4})+D(met_{4})+Q(met_{4})+G(met_{4})+A(met_{4})+H(met_{4})\right] \\ +\Delta met_{4}(t)+met_{6}(t)\times H(met_{6}) \end{matrix}$$

Change in time for IDUs on methadone with symptomatic HIV getting treatment with ART:

$$\frac{dmet_{5}(t)}{dt}=met_{3}(t)\times H(met_{3})-met_{5}(t)\left[M(met_{5})+D(met_{5})+Q(met_{5})+G(met_{5})+P(met_{5})+H(met_{5})\right]+\Delta met_{5}(t)$$

Change in time for IDUs on methadone with AIDS receiving ART:

$$\begin{matrix} \frac{dmet_{6}(t)}{dt}=met_{5}(t)\times P(met_{5})-met_{6}(t)\left[M(met_{6})+D(met_{6})+Q(met_{6})+G(met_{6})+A(met_{6})+H(met_{6})\right] \\ +\Delta met_{6}(t)+met_{4}(t)\times H(met_{4}) \end{matrix}$$

Change in time for non-IDUs with asymptomatic HIV:

$$\frac{dnon_{2}(t)}{dt}=non_{1}(t)\left[\sum_{j=2}^{6} \sum_{Y\in\left\{op,met,non\right\}} C(non_{1},Y_{j})(t)\right]-non_{2}(t)\left[M(non_{2})+D(non_{2})+P(non_{2})\right]+met_{2}(t)\times G(met_{2})$$

Change in time for non-IDUs with symptomatic HIV not treated with ART:

$$\begin{matrix} \frac{dnon_{3}(t)}{dt}=non_{2}(t)\times P(non_{2})-non_{3}(t)\left[M(non_{3})+D(non_{3})+P(non_{3})+H(non_{3})\right] \\ +met_{3}(t)\times G(met_{3})+non_{5}(t)\times H(non_{5}) \end{matrix}$$

Change in time for non-IDUs with AIDS not receiving ART:

$$\begin{matrix} \frac{dnon_{4}(t)}{dt}=non_{3}(t)\times P(non_{3})-non_{4}(t)\left[M(non_{4})+D(non_{4})+A(non_{4})+H(non_{4})\right] \\ +met_{4}(t)\times G(met_{4})+non_{6}(t)\times H(non_{6}) \end{matrix}$$

Change in time for non-IDUs with symptomatic HIV on ART:

$$\frac{dnon_{5}(t)}{dt}=non_{3}(t)\times H(non_{3})-non_{5}(t)\left[M(non_{5})+D(non_{5})+P(non_{5})+H(non_{5})\right]+met_{5}(t)\times G(met_{5})$$

Change in time for non-IDUs with AIDS receiving ART:

$$\begin{matrix} \frac{dnon_{6}(t)}{dt}=non_{5}(t)\times P(non_{5})-non_{6}(t)\left[M(non_{6})+D(non_{6})+A(non_{6})+H(non_{6})\right] \\ +met_{6}(t)\times G(met_{6})+non_{4}(t)\times H(non_{4}) \end{matrix}$$

**Health outcomes and costs**

We computed the total costs in US dollars and benefits measured in QALYs over 20 years, discounted to the present using a 3% annual interest rate, for the status quo and all the considered strategies. Incremental cost-effectiveness ratios (ICERs) were computed by dividing incremental costs by incremental QALYs gained:

*ICER*_strategy A_=$\left[\frac{TotalCost_{\text{ Strategy} \text{A}}-TotalCost_{\text{Reference} \text{Strategy}}}{QALYs_{\text{Strategy} \text{A}}-QALYs_{\text{Reference} \text{Strategy}}}\right]$

HIV prevalence was estimated for the total population

*HIV Prevalence* _total_ *=* $\left[\frac{\sum_{i=2}^{6} \sum_{X\in\left\{op,met,non\right\}} X_{i}(t)}{\sum_{i=1}^{6} \sum_{X\in\left\{op,met,non\right\}} X_{i}(t)}\right]$ ,

the IDU population

*HIV Prevalence* _IDU_ *=* $\left[\frac{\sum_{i=2}^{6} \sum_{X\in\left\{op,met\right\}} X_{i}(t)}{\sum_{i=1}^{6} \sum_{X\in\left\{op,met\right\}} X_{i}(t)}\right]$ ,

and the non-IDU population

*HIV Prevalence* _non-IDU_ *=* $\left[\frac{\sum_{i=2}^{6} non_{i}(t)}{\sum_{i=1}^{6} non_{i}(t)}\right]$ .

In order to compute the number of infections averted, we computed for each strategy and the status quo the number of new infections occurring at each time *t*. The number of infections averted by a strategy is the difference between the total number of new infections under the strategy, and the total number of new infections in the status quo.

**One-way sensitivity analyses**

We performed one-way sensitivity analysis on all model parameters, and found that our results are robust under a wide range of assumptions, with the “high methadone” and “high treatment” strategies as the preferred options. Table S5 and Figure S2 summarize the results of the cost-effectiveness analysis for the “high methadone” strategy, for low and high values of the 20 parameters that have the greatest effect on the incremental cost-effectiveness ratio. In all of these scenarios, “high methadone” remained the most cost-effective alternative. The results were most sensitive to the preference of IDUs for other IDUs as sexual partners, the costs of the methadone regimen, the effectiveness of methadone in reducing needle sharing, HIV prevalence in the population, and non-HIV medical costs.

**Probabilistic sensitivity analysis**

We performed a probabilistic sensitivity analysis (PSA) using a second-order Monte Carlo simulation with 1000 runs. We sampled all parameters from beta distributions scaled according to the best information available from the literature about the mean and range of the parameters (Table S3). We used parameters that yielded a narrower distribution when there is more certainty about the mean estimate, and broader distributions when the mean value is less certain. The distributions chosen for key model parameters are described below.

*Percentage of sexual partners of IDUs who are also IDUs:* Given the difficulty of estimating behavioral parameters and the importance of IDU to non-IDU sexual transmission in moving the epidemic to the general population, we varied the value of this parameter between 20%-70% with a mean of 45% [10,12,24,33,40]. For the PSA we used a beta distribution with a mean of 45 and a standard deviation of 8.333.

*Methadone effectiveness in reducing risky injection behavior*: There is uncertainty regarding the effectiveness of methadone in reducing risky injection behavior in Ukraine, given the substances injected by IDUs and the behavioral characteristics of IDUs. Consistent with values from the literature [11,20,38] and from the pilot programs on substitution therapy in Ukraine [46], we assumed a range of 60%-99% with a mean of 85%. For the PSA we used a beta distribution with a mean of 85 and a standard deviation of 6.953.

*HIV prevalence*: For overall HIV prevalence in adults 15-49, we assumed the 95% CI was approximately 0.95%-2.37%, with a mean of 1.63% [3,4]. In the PSA, we used a beta distribution with mean 1.63 and standard deviation 0.1085. The 95% CI for prevalence of HIV in IDUs is 17.3%-70%, with a mean of 41.2% [3,4]. We used a beta distribution with mean 41.2 and standard deviation 3.9067 for our analysis. The estimates for the proportion of IDUs in the population range from 1.34% to 1.75%, with a mean of 1.60% [4]. For the PSA, we used a beta distribution with mean 1.60 and standard deviation 0.0731. These distributions reflect the information available from the literature about the certainty of the parameters.

*ART reduction of infectivity*: Given the uncertainty in the effects of ART on needle-related infectivity, we assumed a range of 10%-90% for the reduction in infectivity if on ART, with a mean of 50% [33]. In the PSA we modeled this parameter using a beta distribution with mean 50 and standard deviation 15.12. For the reduction in sexual infectivity given by ART, we assumed a range of 50%-99% with a mean of 90% [27,33,34]. We used a beta distribution with mean 90 and standard deviation 5.774.

**REFERENCES (PAPER AND APPENDIX)**

1. Mathers BM, Degenhardt L, Phillips B, Wiessing L, Hickman M, et al. (2008) Global epidemiology of injecting drug use and HIV among people who inject drugs: a systematic review. The Lancet 372: 1733-1745.

2. Celentano DD, Beyrer C, Wolfe D, Elovich R, Boltaev A, et al. (2008) HIV in Central Asia: Tajikistan, Uzbekistan and Kyrgyzstan. Public Health Aspects of HIV/AIDS in Low and Middle Income Countries: Springer New York. pp. 557-581.

3. Joint United Nations Programme on HIV/AIDS (UNAIDS) (2008) **Ukraine - National report on monitoring progress towards the UNGASS declaration of commitment on HIV/AIDS**. Geneva: United Nations.

4. Kruglov YV, Kobyshcha YV, Salyuk T, Varetska O, Shakarishvili A, et al. (2008) The most severe HIV epidemic in Europe: Ukraine's national HIV prevalence estimates for 2007. Sex Transm Infect 84: i37-41.

5. Hamers FF, Downs AM (2003) HIV in central and eastern Europe. Lancet 361: 1035-1044.

6. Kelly JA, Amirkhanian YA (2003) The newest epidemic: a review of HIV/AIDS in Central and Eastern Europe. Int J STD AIDS 14: 361-371.

7. DeBell D, Carter R (2005) Impact of transition on public health in Ukraine: case study of the HIV/AIDS epidemic. BMJ 331: 216-219.

8. Saad MD, Shcherbinskaya AM, Nadai Y, Kruglov YV, Antonenko SV, et al. (2006) Molecular epidemiology of HIV Type 1 in Ukraine: birthplace of an epidemic. AIDS Res Hum Retroviruses 22: 709-714.

9. Barnett T, Whiteside A, Khodakevich L, Kruglov Y, Steshenko V (2000) The HIV/AIDS epidemic in Ukraine: its potential social and economic impact. Soc Sci Med 51: 1387-1403.

10. Barcal K, Schumacher J, Dumchev K, Moroz L (2005) A situational picture of HIV/AIDS and injection drug use in Vinnitsya, Ukraine. Harm Reduct J 2: 16.

11. Bruce RD, Dvoryak S, Sylla L, Altice FL (2007) HIV treatment access and scale-up for delivery of opiate substitution therapy with buprenorphine for IDUs in Ukraine--programme description and policy implications. Int J Drug Policy 18: 326-328.

12. Booth RE, Kwiatkowski CF, Brewster JT, Sinitsyna L, Dvoryak S (2006) Predictors of HIV sero-status among drug injectors at three Ukraine sites. AIDS 20: 2217-2223.

13. Joint United Nations Programme on HIV/AIDS (UNAIDS) (2008) **Ukraine - Country situation factsheet**. Geneva: United Nations.

14. Open Society Institute & Soros Foundation Network (2007) **Newsflash Ukraine: Methadone scale up authorized**. New York, NY: Open Society Institute.

15. Joint United Nations Programme on HIV/AIDS (UNAIDS) (2008) **Progress towards universal access - Ukraine factsheet**. Geneva: United Nations.

16. UNAIDS/WHO Working Group on Global HIV/AIDS and STI Surveillance (2008) **Epidemiological Factsheet on HIV and AIDS: Core Data on Epidemiology and Response - Ukraine**. Geneva: World Health Organization (WHO) and Joint United Nations Programme on HIV/AIDS (UNAIDS).

17. International HIV/AIDS Alliance in Ukraine (2008) **International HIV/AIDS Alliance in Ukraine: 2007 Annual Report**. Kyiv, Ukraine: International HIV/AIDS Alliance in Ukraine.

18. Joint United Nations Programme on HIV/AIDS (UNAIDS) (2007) **UNGASS - National Composite Policy Index (NCPI) 2007 - Ukraine**. Geneva: United Nations.

19. Schleifer R (2006) **Rhetoric and Risk: Human Rights Abuses Impeding Ukraine’s Fight against HIV/AIDS**. New York, NY: Human Rights Watch.

20. Connock M, Juarez-Garcia A, Jowett S, Frew E, Liu Z, et al. (2007) **Methadone and buprenorphine for the management of opioid dependence: a systematic review and economic evaluation**. Southampton, United Kingdom: National Institute for Health Research - Health Technology Assessment Programme.

21. Marsch LA (1998) The efficacy of methadone maintenance interventions in reducing illicit opiate use, HIV risk behavior and criminality: a meta-analysis. Addiction 93: 515-532.

22. Schilling R, Dornig K, Lungren L (2006) Treatment of heroin dependence: effectiveness, costs, and benefits of methadone maintenance. Res Social Work Practice 16: 48-56.

23. Zaric GS, Brandeau ML, Barnett PG (2000) Methadone maintenance and HIV prevention: a cost-effectiveness analysis. Manage Sci 46: 1013-1031.

24. Vickerman P, Kumaranayake L, Balakireva O, Guinness L, Artyukh O, et al. (2006) The cost-effectiveness of expanding harm reduction activities for injecting drug users in Odessa, Ukraine. Sex Transm Dis 33: S89-S102.

25. Panel on Antiretroviral Guidelines for Adults and Adolescents (2008) **Guidelines for the use of antiretroviral agents in HIV-1-infected adults and adolescents**. Bethesda, MD: United States Department of Health and Human Services.

26. World Health Organization (WHO) (2006) **Antiretroviral Therapy for HIV Infection in Adults and adolescents: Recommendations for a Public Health Approach - 2006 Revision**. Geneva: World Health Organization.

27. Castilla J, del Romero J, Hernando V, Marincovich B, Garcia S, et al. (2005) Effectiveness of highly active antiretroviral therapy in reducing heterosexual transmission of HIV. J AIDS 40: 96-101.

28. Egger M, May M, Chêne G, Phillips AN, Ledergerber B, et al. (2002) Prognosis of HIV-1-infected patients starting highly active antiretroviral therapy: a collaborative analysis of prospective studies. Lancet 360: 119-129.

29. Grabar S, Moing VL, Goujard C, Leport C, Kazatchkine MD, et al. (2000) Clinical outcome of patients with HIV-1 infection according to immunologic and virologic response after 6 months of highly active antiretroviral therapy. Ann Intern Med 133: 401-410.

30. Royce RA, Sena A, Cates W, Cohen MS (1997) Sexual transmission of HIV. N Engl J Med 336: 1072-1078.

31. Wood E, Hogg RS, Yip B, Harrigan PR, O'Shaughnessy MV, et al. (2003) Effect of medication adherence on survival of HIV-infected adults who start highly active antiretroviral therapy when the CD4+ cell count is 0.200 to 0.350 x 10^9^ cells/L. Ann Intern Med 139: 810-816.

32. Kaplan EH, Heimer R (1992) A model-based estimate of HIV infectivity via needle sharing. J AIDS 5: 1116-1118.

33. Long EF, Brandeau ML, Galvin CM, Vinichenko T, Tole SP, et al. (2006) Effectiveness and cost-effectiveness of strategies to expand antiretroviral therapy in St. Petersburg, Russia. AIDS 20: 2207-2215.

34. Sanders GD, Bayoumi AM, Sundaram V, Bilir SP, Neukermans CP, et al. (2005) Cost-effectiveness of screening for HIV in the era of highly active antiretroviral therapy. N Engl J Med 352: 570-585.

35. International HIV/AIDS Alliance in Ukraine (2007) **Report on Implementing the Programme supported by the Global Fund to Fight AIDS, Tuberculosis and Malaria in Ukraine**. Kyiv, Ukraine: International HIV/AIDS Alliance in Ukraine.

36. International HIV/AIDS Alliance in Ukraine (2008) **Methadone substitution therapy starts in Ukraine**. Kyiv, Ukraine: International HIV/AIDS Alliance in Ukraine.

37. Grönbladh L, Gunne LM (1989) Methadone-assisted rehabilitation of Swedish heroin addicts. Drug and Alcohol Dependence 24: 31-37.

38. Zaric G, Barnett P, Brandeau M (2000) HIV transmission and the cost-effectiveness of methadone maintenance. Am J Public Health 90: 1100-1111.

39. Booth RE, Kwiatkowski CF, Mikulich-Gilbertson SK, Brewster JT, Salomonsen-Sautel S, et al. (2006) Predictors of risky needle use following interventions with injection drug users in Ukraine. Drug Alcohol Depend 82: S49-S55.

40. Booth RE, Mikulich-Gilbertson SK, Brewster JT, Salomonsen-Sautel S, Semerik O (2004) Predictors of Self-Reported HIV Infection Among Drug Injectors in Ukraine. JAIDS Journal of Acquired Immune Deficiency Syndromes 35: 82-88.

41. European Monitoring Centre for Drugs and Drug Addiction (EMCDDA) (2008) Country overview: Ukraine. Lisbon, Portugal: European Monitoring Centre for Drugs and Drug Addiction.

42. Liu C, Ostrow D, Detels R, Hu Z, Johnson L, et al. (2006) Impacts of HIV infection and HAART use on quality of life. Qual Life Res 15: 941-949.

43. Gill CJ, Griffith JL, Jacobson D, Skinner S, Gorbach SL, et al. (2002) Relationship of HIV viral loads, CD4 counts, and HAART use to health-related quality of life. J AIDS 30: 485-492.

44. Mannheimer SB, Matts J, Telzak E, Chesney M, Child C, et al. (2005) Quality of life in HIV-infected individuals receiving antiretroviral therapy is related to adherence. AIDS Care 17: 10-22.

45. Jelsma J, MacLean E, Hughes J, Tinise X, Darder M (2005) An investigation into the health-related quality of life of individuals living with HIV who are receiving HAART. AIDS Care 17: 579 - 588.

46. Dvoriak S. **Opioid Substitution Therapy (OST) with Buprenorphine in Ukraine - Way to Prevent HIV/AIDS among IDUs**; 2007; Sydney, Australia.

47. World Health Organization (WHO) (2005) **Summary Country Profile for HIV/AIDS Treatment Scale-Up - Ukraine**. Geneva: World Health Organization.

48. World Health Organization (WHO) (2009) Global Price Reporting Mechanism. Geneva: WHO.

49. World Health Organization (WHO) (2003) **Making Choices in Health: WHO Guide to Cost-Effectiveness Analysis**. Geneva: World Health Organization.

50. Hutubessy R, Chisholm D, Edejer TT (2003) Generalized cost-effectiveness analysis for national-level priority-setting in the health sector. Cost Eff Resour Alloc 1: 8.

51. Weinstein MC, Stason WB (1977) Foundations of Cost-Effectiveness Analysis for Health and Medical Practices. New England Journal of Medicine 296: 716-721.

52. Gold M, Siegel J, Russell L, Weinstein M (1996) Cost-Effectiveness in Health and Medicine. New York: Oxford University Press.

53. Gold MR, Stevenson D, Fryback DG (2002) HALYs and QALYs and DALYs, Oh My: similarities and differences in summary measures of population health. Annu Rev Public Health 23: 115-134.

54. Murray CJL, Salomon JA, Mathers CD, Lopez AD, editors (2002) Summary Measures of Population Health. Concepts, Ethics, Measurement and Applications. Geneva: World Health Organization.

55. Sassi F (2006) Calculating QALYs, comparing QALY and DALY calculations. Health Policy Plan 21: 402-408.

56. Pinkerton SD, Abramson PR (1997) Effectiveness of condoms in preventing HIV transmission. Soc Sci Med 44: 1303-1312.

57. Feshbach M, Galvin C (2005) **HIV/AIDS in Ukraine - An Analysis of Statistics**. Washington, DC: Woodrow Wilson International Center for Scholars.

58. Grönbladh L, Öhlund LS, Gunne LM (1990) Mortality in heroin addiction: impact of methadone treatment. Acta Psychiatr Scand 82: 223-227.

59. Smyrnov P (2007) **Making the change: 3 years of Global Fund supported treatment in Ukraine - Evidence versus policy**. Tbilisi, Georgia: International HIV/AIDS Alliance in Ukraine.

60. The World Bank (2006) S**ocioeconomic Impact of HIV/AIDS in Ukraine**. Washington, DC: World Bank.

61. Tengs TO, Lin TH (2002) A meta-analysis of utility estimates for HIV/AIDS. Med Decis Making 22: 475-481.

62. Lucas GM, Mullen BA, Weidle PJ, Hader S, McCaul ME, et al. (2006) Directly administered antiretroviral therapy in methadone clinics is associated with improved HIV treatment outcomes, compared with outcomes among concurrent comparison groups. Clin Infect Dis 42: 1628-1635.
